# A comprehensive review of transcription factor-mediated regulation of secondary metabolites in plants under environmental stress

**DOI:** 10.1007/s44154-024-00201-w

**Published:** 2025-02-24

**Authors:** Karim Rabeh, Mohamed Hnini, Malika Oubohssaine

**Affiliations:** 1 Oasis System Research Unit, Regional Center of Agricultural Research of Errachidia, National Institute of Agricultural Research, Rabat, PO. Box 415, 10090 Morocco; 2 Microbiology and Molecular Biology Team, Center of Plant and Microbial Biotechnologies, Biodiversity and Environment, Faculty of Sciences, Mohammed V University, Rabat, Morocco; 3 High School of Technology Laayoune, Ibn Zohr University, Agadir, Morocco

**Keywords:** Plant secondary metabolites, Transcription factors, Abiotic stress, Biotic stress

## Abstract

Plants encounter a wide range of biotic and abiotic environmental stresses throughout their lifespan. To adapt and survive, they have evolved complex defense mechanisms that involve the production of secondary metabolites (SMs), which play critical roles in enhancing plant resilience. These bioactive compounds function as deterrents to herbivores, barriers against pathogens, and protectants against oxidative stress. Plants regulate the synthesis and accumulation of secondary metabolites (SMs) in response to various environmental factors such as drought, salinity, cold, heat, heavy metals, and UV-B radiation. These stress conditions can significantly alter SM levels as part of the plant's adaptive response. Transcription factors (TFs) are essential mediators in this process, regulating gene expression involved in SM biosynthesis. By interacting with various signaling pathways, TFs fine-tune the plant's defense mechanisms, ensuring the timely accumulation of specific SMs that mitigate stress impacts. This review provides a comprehensive examination of the influence of environmental factors on SM accumulation in plants under both biotic and abiotic stress conditions. Additionally, we thoroughly discuss the roles of key TFs—including AP2/ERF, WRKY, bHLH, bZIP, MYB, and NAC—in regulating SM biosynthesis, highlighting their contributions to the plant's ability to withstand and adapt to stress.

**Supplementary Information:**

The online version contains supplementary material available at 10.1007/s44154-024-00201-w.

## Introduction

Plants, as sessile organisms, are continuously exposed to a wide range of environmental factors, including extreme temperatures, drought, salinity, pollutants, and pathogens (Bellucci et al. 2024). These stressors pose significant challenge plant growth, development, and productivity, thereby threatening global food security and ecosystem stability. In response, plants have evolved robust and complex innate immune systems over time to adapt to varying environmental conditions. Throughout their lifespan, plants synthesize both primary and secondary metabolites (PMs and SMs). While PMs are essential for fundamental processes such as growth and development, SMs, which are natural byproducts of primary metabolic processes, play crucial roles in enabling plants to cope with unfavorable environmental conditions (Pagare et al. 2015; Jha and Mohamed 2022).

Over 200,000 SMs from plants have been identified and characterized (Satish et al. 2020). These SMs can be categorized into four major groups based on their biosynthetic pathways: terpenes (including diterpenes, sesquiterpenes, triterpenes, sesquaterpenes, and saponins) (Pazouki and Niinemets 2016), phenolics (such as flavonoids, lignins, coumarins, and tannins) (Deng and Lu 2017), sulfur-containing compounds (like glutathione, phytoalexins, and glucosinolates) (Noctor et al. 2012), and nitrogen-containing compounds (including alkaloids, cyanogenic glucosides, and non-protein amino acids) (Upadhyay et al. 2024).

The essential roles of plant SMs involve providing diverse protective mechanisms against pathogens, insects, and predators, largely due to their toxic properties (Divekar et al. 2022). Additionally, certain plant SMs facilitate communication with microorganisms, enhancing tolerance to biotic stress, and mitigating the effects of abiotic stress factors such as drought, salinity, extreme temperatures, and UV radiation (Yeshi et al. 2022; Jha and Mohamed 2022). Under stress conditions, these metabolites often accumulate to higher levels, acting as signaling molecules that trigger the upregulation of defense-related genes. Thus, plant SMs are vital for enhancing plant resilience under adverse conditions. Plants employ a range of defense strategies at the genetic and molecular levels, involving various genes and TFs (Quintin et al. 2014; Lamalakshmi Devi et al. 2017; Li et al. 2022). Through specialized sensors and receptors, plants can detect threats and initiate defense responses. TFs are crucial in these signaling pathways, as they interpret stress signals and regulate the expression of downstream defense-related genes (Ng et al. 2018; Meraj et al. 2020). TFs such as AP2/ERF, WRKY, bHLH, bZIP, MYB, and NAC AP2/ERF, WRKY, bHLH, bZIP, MYB, and NAC have been found to participate in the regulation of SM biosynthesis (Patra et al. 2013).

This review aims to provide a comprehensive overview of plant secondary metabolites, emphasizing their roles in abiotic and biotic stress. Furthermore, it presents a thorough perspective on plant stress responses involving secondary metabolism regulated by TFs. The interplay between TFs and their associated SMs is highlighted, underscoring their pivotal role in enhancing plant resilience through the mediation of secondary metabolite biosynthesis and accumulation.

## Environment stress effects on plant SMs

Plants, due to their sessile nature, are exposed to a broad spectrum of environmental stresses throughout their lifespan. These stresses induce the production of reactive oxygen species (ROS), which can damage vital cellular components, including proteins, nucleic acids, and lipids, ultimately threatening cell viability. To mitigate ROS accumulation and ensure survival, plants have evolved a complex antioxidative defense system composed of enzymatic and non-enzymatic molecules. A significant portion of these defense molecules belongs to the category of secondary metabolites (SMs), which play a key role in stress responses (Bartwal et al. 2013).

In response to various abiotic and biotic stresses, such as drought, salinity, herbivory, and pathogenic attacks, plants synthesize a diverse array of SMs as part of their adaptive defense mechanisms. As shown in Tables S1-S5 and S6, the levels of specific SMs fluctuate depending on the type of abiotic or biotic stress encountered. These dynamic changes in SM composition highlight their critical role in helping plants cope with environmental challenges.

### Abiotic stress

#### Drought stress

Drought stress profoundly affects SM production in plants, driving a range of adaptive strategies that vary among species. Under drought conditions, plants often increase the synthesis of SMs as part of their stress response and defense mechanisms. This stress also induces changes in metabolic pathways, leading to alterations in the abundance of specific compounds. The impact of drought on metabolite production varies by species, reflecting diverse adaptive strategies (Table S1).

##### Flavonoids and terpenoids

*Ocimum basilicum* (basil) responds to drought conditions by increasing the production of flavonoids such as methyl chavicol and methyleugenol, indicating a protective role of these compounds against drought-induced oxidative stress (Abdollahi Mandoulakani et al. 2017). In a similar manner, *Salvia dolomitica* exhibits a rise in sesquiterpenes, compounds renowned for their defensive and stress-tolerant properties (Caser et al. 2019). Likewise, *Vitex agnus-castus* adapts to drought by elevating levels of terpenoids such as α-pinene and β-terpinyl acetate, which potentially enhance its defense mechanisms (Rezaei et al. 2019).

##### Phenolic compounds

Increased production of phenolic compounds is observed in *Lallemantia* sp., which may help mitigate drought stress effects (Jamal Omidi et al. 2018). *Crataegus laevigata* and *C. monogyna* also shows elevated levels of chlorogenic acid, catechin, and ( −)-epicatechin, which contribute to the plant's antioxidant defense system during drought (Kirakosyan et al. 2004).

##### Alkaloids

Under drought stress, *Glycine max* (soybean) and *Papaver somniferum* (poppy) exhibit increased alkaloid levels, including trigonelline in soybean (Cho et al. 2003) and morphine and codeine in poppy (Szabó et al. 2005). These alkaloids may play a role in modulating growth and stress responses.

##### Monoterpenes and glycosides

*Eucomis autumnalis* and *Scrophularia ningpoensis* show an increase in iridoid monoterpenes, which possess anti-inflammatory and antioxidant properties, aiding drought survival (Masondo et al. 2014), While *Scrophularia ningpoensis* also demonstrates a selective increase in glycosides like catalpol and aucubin, it shows a decrease in others, reflecting a nuanced stress response (Wang et al. 2010).

#### Salt stress

Plants exhibit complex mechanisms of SM production in response to salt stress, highlighting species-specific adaptation strategies. When exposed to high concentrations of sodium (Na^+^) and chloride (Cl^−^), plants experience disruptions in osmotic balance and ionic homeostasis. To mitigate these effects, plants sequester toxic ions in vacuoles and synthesize compatible solutes. SMs, such as phenolics, flavonoids, and alkaloids, play crucial roles in reducing salt-induced oxidative stress and facilitating osmotic adjustment (Isah 2019). The production of these metabolites often increases under saline conditions, serving as protective agents (Table S2). Each plant species exhibits unique patterns of SM production under salt stress, reflecting their evolutionary adaptations (Kumar et al. 2023).

##### Phenolic compounds and flavonoids

*Limonium bicolor* experiences a decrease in several phenolic compounds and flavonoids under salt stress, suggesting compromised antioxidant defenses (Wang et al. 2016d). Conversely, *Ocimum basilicum* shows a mixed response, with decreases in eugenol and methyl eugenol but increases in terpenoids and phenolic acids like linalool and caffeic acid, possibly reallocating resources to more effective stress countermeasures (Bahcesular et al. 2020).

##### Terpenoids and monoterpenes

*Mentha spicata* increases carvone production in response to salt stress, indicating its stress-protective role. However, limonene levels decrease, suggesting a shift in metabolic priorities (Chrysargyris et al. 2019). *Origanum majorana* displays variable responses, with a decrease in trans-hydrate sabinene and terpinen-4-ol but an increase in terpinene-4-ol and other phenolic compounds, reflecting complex metabolic adjustments (Baâtour et al. 2011; Jelali et al. 2011).

##### Quaternary ammonium compounds and related enzyme activity

*Triticum aestivum* increases betaine aldehyde dehydrogenase activity, reducing membrane permeability and oxidative damage under salt stress (Yadav et al. 2017) while, *Helianthus annuum* enhances phenolic acid HaHQT2 activity to counteract hydrogen peroxide-induced oxidative stress (Cheevarungnapakul et al. 2019).

#### Cold and heat stress

Cold and heat stress prompt specific adjustments in SM production, showcasing how plants adapt to extreme temperatures. Cold stress, particularly at temperatures between 4–10 °C, triggers the enhanced accumulation of flavonoids and terpenoids, driven by shifts in endogenous plant hormones like jasmonic acid and abscisic acid. Additionally, cold stress promotes the production of phenolic compounds, which integrate into plant cell walls as suberin or lignin, bolstering structural integrity and protecting against freezing. Cryoprotective compounds, such as low-molecular-weight nitrogenous substances, including alkaloids, sesquiterpenes, isoflavones, and non-protein amino acids, are also synthesized to mitigate damage from freezing conditions (He et al. 2023).

Conversely, heat stress alters SM profiles, often increasing the production of specific phenylpropanoids as a defense against oxidative stress caused by elevated temperatures. Plants also enhance antioxidant production in response to heat stress, crucial for neutralizing reactive oxygen species (ROS) and maintaining cellular integrity. Species-specific adaptations to heat stress result in varied responses, reflecting evolutionary strategies to manage extreme temperatures while sustaining physiological functions (Commisso et al. 2016).

As mentioned in Table S3, *Cyanea acuminata* increases 10-hydroxy camptothecin production under this stress, potentially aiding its defense (Wang et al. 2003). *Catharanthus roseus* exhibits increased levels of vindoline and catharanthine, key alkaloids linked to stress resistance (Guo et al. 2007). However, a decrease in vindoline under some conditions suggests variability based on experimental parameters (Dutta et al. 2007). *Quercus rubra*enhances isoprene production, which protects photosynthetic processes under cold (Hanson and Sharkey 2001). *Brassica oleracea* boosts quercetin levels to mitigate oxidative stress (Mølmann et al. 2015). *Daucus carota* increases α-farnesene production, assisting in cold acclimation (Helmig et al. 2007).

*Cucumis acuminatus* enhances the production of 10-hydroxycamptothecin when exposed to heat stress, indicating its potential role in promoting heat tolerance*.* (Yuan-Gang et al. 2003). *Daucus carota* shows a decrease in α-terpinolene but an increase in α-caryophyllene and β-farnesene, reflecting a dynamic adjustment to heat stress (Rosenfeld et al. 2002). *Quercus rubra* enhances isoprene emissions, protecting against heat-induced damage (Hanson and Sharkey 2001). *Solanum lycopersicon* reduces α-humulene levels, indicating a resource reallocation under high temperatures (Copolovici et al. 2012). *Centella asiatica* increases asiaticoside production, potentially protecting against heat damage (Randriamampionona et al. 2007). *Daucus carota* also increases anthocyanins, coumaric acid, and caffeic acid to aid in heat stress mitigation (Commisso et al. 2016).

#### Heavy metal stress

Heavy metals impact SM production in plants, leading to complex adaptive responses. Exposure to these metals can either enhance or inhibit metabolite synthesis, reflecting the plants' strategies to counteract metal toxicity. Heavy metal stress often induces the production of SMs like phenolics, flavonoids, and alkaloids, which play crucial roles in detoxification and protection against oxidative damage. Conversely, excessive metal contamination can suppress metabolite production by impairing plant growth and metabolism. Additionally, the oxidative stress caused by heavy metals generates reactive oxygen species (ROS), which activate signaling pathways that can further influence metabolite synthesis. The response to heavy metal stress varies across plant species, highlighting the need for species-specific insights into how plants adapt to these environmental challenges.

The impact of heavy metals on SM production in plants showcases a range of adaptive responses (Table S4). *Chrysopogon zizanioides* enhances its production of phenolic compounds when exposed to arsenic (As), chromium (Cr), copper (Cu), nickel (Ni), lead (Pb), and zinc (Zn), which helps the plant detoxify and manage metal stress (Melato et al. 2012). In contrast, *Gynura procumbens* shows a reduction in phenolics, flavonoids, and total saponin content under cadmium (Cd) and copper (Cu) stress, indicating a negative effect on its metabolic profile and medicinal properties (Ibrahim et al. 2017). Similarly, *Drimia elata* experiences decreased levels of total phenolics and flavonoids when subjected to cadmium (Cd) and aluminum (Al), pointing to compromised antioxidant capacity (Okem et al. 2015). *Phyllanthus amarus* also exhibits a reduction in lignans like phyllanthin and hypophyllanthin under cadmium stress, potentially affecting its defense mechanisms and therapeutic value (Rai et al. 2005). On the other hand, *Malus domestica, Phaseolus vulgaris*, and *Triticum aestivum* show increased levels of caffeic acid equivalents under zinc (Zn) deficiency, suggesting a compensatory response to enhance stress tolerance (Zhang et al. 1991). *Kandelia obovata* exhibits increased production of protocatechuic, ferulic, and cinnamic acids in response to cadmium (Cd) and zinc (Zn) toxicity, likely facilitating detoxification and stress mitigation (Chen et al. 2020). Conversely, *Imperata condensata* demonstrates a decrease in catechin levels under copper (Cu) stress, indicative of suppressed antioxidant defenses (Meier et al. 2012). In contrast, *Helianthus annuus* increases coumaric acid production under copper stress, suggesting an adaptive strategy to counteract oxidative stress induced by copper ions (Meier et al. 2012). Finally, *Zea mays* enhances the synthesis of flavonoids and phenolic compounds, including catechin, catechol, curcumin, and quercetin, under aluminum (Al) toxicity, thereby boosting its protective mechanisms against oxidative damage (Kidd et al. 2001). While aluminum is not classified as a heavy metal, it is a toxic light metal that can cause significant stress in plants under acidic soil conditions.

#### UV-B stress

Ultraviolet-B (UV-B) radiation significantly influences SM production in plants, with responses ranging from increased to decreased metabolite levels. UV-B acts as an abiotic elicitor, stimulating the production of SMs like flavonoids, phenolics, and alkaloids, which are crucial for plant defense against UV-B-induced damage and oxidative stress. These metabolites provide photoprotection by absorbing UV-B light, thereby reducing cellular damage. The response to UV-B varies across plant species; some may show a marked increase in protective metabolites, while others exhibit minimal changes. In medicinal plants, UV-B exposure can enhance levels of bioactive compounds, contributing to their therapeutic properties. However, excessive UV-B radiation can suppress overall plant growth and metabolic activity, leading to reduced SM production and increased oxidative stress.

UV-B radiation induces specific adjustments in SM production across various plant species (Table S5). For instance, *Catharanthus roseus*, renowned for its medicinal properties, increases the production of alkaloids such as catharanthine in response to UV-B stress, reflecting a defensive strategy to enhance resilience (Ramani and Chelliah 2007). Other studies have reported an overall increase in SMs in *Catharanthus roseus* under UV-B radiation, though specific metabolite classes were not detailed (Schluttenhofer et al. 2014). This broad enhancement of SMs as a response to UV-B radiation is echoed in other species. For example, *Artemisia annua*, famed for its medicinal value, responds to UV-B exposure with increased artemisinin production, highlighting its ability to upregulate crucial metabolites for environmental stress adaptation (Mehrotra et al. 2018). Similarly, *Clarkia breweri* boosts the production of phenolic compounds, specifically eugenol, likely enhancing its defensive mechanisms against UV-B stress (Siddiqui et al. 2009). Moreover, *Fagopyrum esculentum* raises quercetin and catechin levels, which are vital flavonoids protecting plant tissues from oxidative damage and UV-B stress (Regvar et al. 2012). Parallel to these findings, *Camptotheca acuminata* exhibits elevated production of phenolics under UV-B stress, reflecting a general enhancement in its SMs profile for UV-B defense (Takshak and Agrawal 2019). In the same vein, *Gnaphalium luteoalbum* shows increased production of the flavonoid calycopterin in response to UV-B exposure, indicating a role for flavonoids in UV-B protection (Cuadra et al. 1997). Likewise, *Gossypium hirsutum* demonstrates increased SM production under UV-B stress, although specific metabolite classes were not specified (Cuadra 2015). Furthermore, *Hordeum vulgare* enhances the production of caponarin and luteolin, two flavonoids, in response to UV-B radiation, contributing to the plant’s defense mechanisms against UV-B-induced oxidative stress (Ra et al. 2020). Finally, *Quercus ilex* increases kaempferol levels under UV-B stress, suggesting its role in protecting the plant from UV-B damage (Skaltsa et al. 1994).

**Fig. 1:**
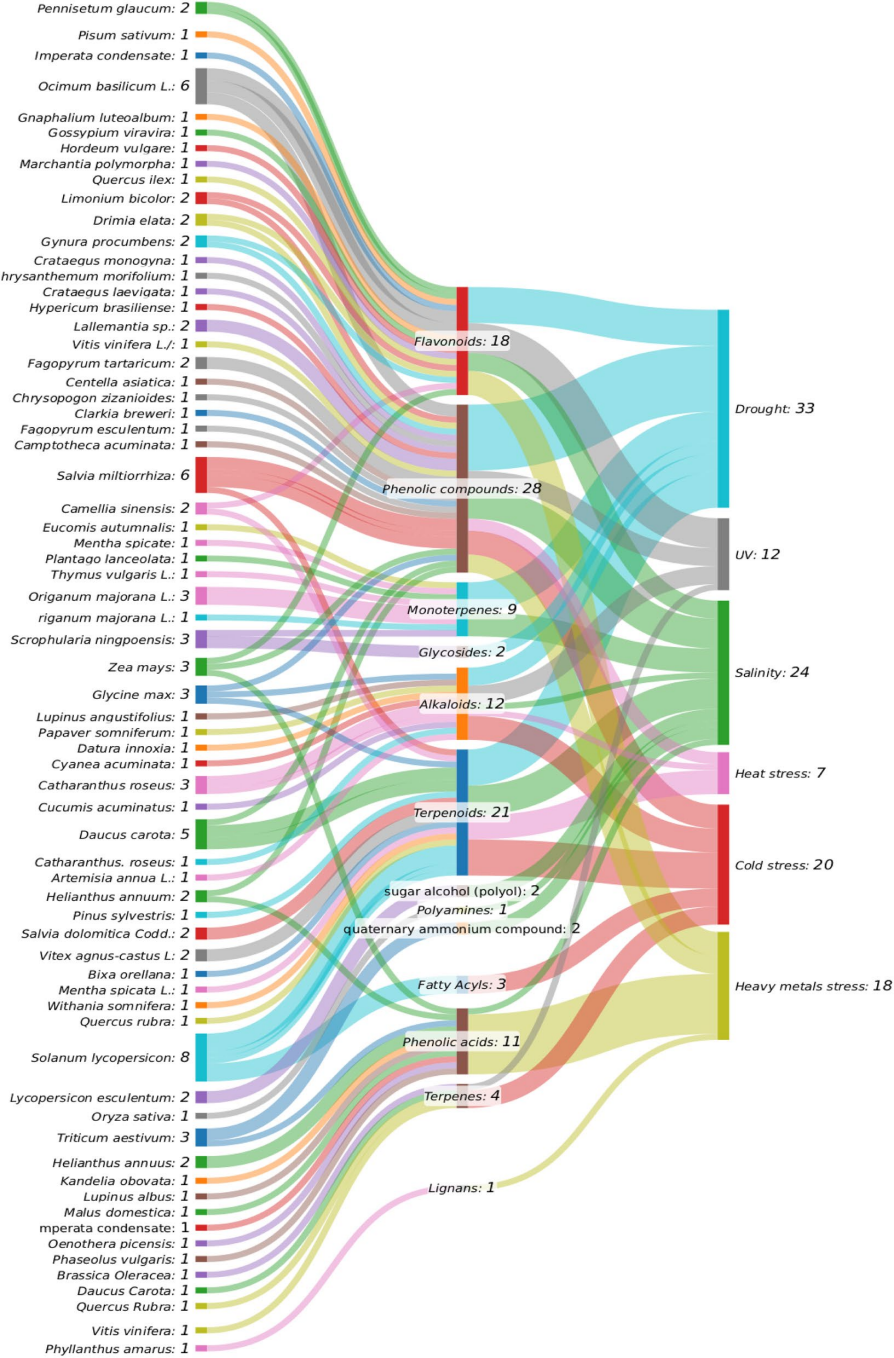
Sankey diagram illustrating the interconnections between plant species, secondary metabolites, and abiotic stress responses. The diagram was created with SankeyMATIC

Overall, sankey diagram illustrates (Fig. 1) the relationships between various plant species, their synthesized secondary metabolites, and the types of abiotic stressors they mitigate. Among the metabolites, flavonoids (18 connections) and phenolic compounds (28 connections) are the most prominent, highlighting their extensive roles in plant defense mechanisms. In particular, the strong link between flavonoids and UV-B stress suggests a significant protective function of these compounds, which are known for absorbing harmful UV radiation and scavenging reactive oxygen species (ROS) produced during stress. Terpenoids (21 connections) and alkaloids (12 connections) also significantly contribute to the plant's response to stress. Drought and salinity emerge as the most prevalent stressors addressed by these metabolites, particularly through the actions of flavonoids, phenolic compounds, and terpenoids.

### Biotic stress

Plant SMs play an essential role in managing biotic stress, offering a sophisticated array of defenses against pests and pathogens. These metabolites operate through both direct and indirect mechanisms, enhancing plant resilience in the face of biotic threats (Table S6).

At the frontline of these defenses are direct mechanisms, where SMs such as phytotoxins and phytoalexins act as potent inhibitors of herbivores and pathogens. For instance, alkaloids, well-known for their toxic properties, serve as effective deterrents against herbivores, preventing damage and feeding. This direct defense is complemented by indirect mechanisms that utilize volatile organic compounds (VOCs) to attract natural enemies of herbivores, such as predators and parasitoids. By promoting the presence of these beneficial organisms, plants effectively manage pest populations, highlighting a sophisticated layer of indirect defense. Furthermore, chemical signaling is integral to the plant's defense strategy. Compounds like salicylic acid and jasmonic acid are pivotal in orchestrating the plant's response to stress. These signaling molecules activate defense genes and stimulate the production of protective metabolites, linking the plant's stress perception to its defensive response.

The diversity of SMs itself emphasizes the complexity of plant defense. Phenolic compounds such as flavonoids and tannins fortify cell walls and deter herbivores, while terpenoids offer repellent and antimicrobial properties. Nitrogen-containing compounds like alkaloids and cyanogenic glycosides provide direct toxicity, and sulfur-containing glucosinolates produce harmful compounds that deter pests. This rich variety of defensive chemicals reflects the plant’s evolutionary adaptations to specific threats.

Table S6 highlights the diverse mechanisms through which SM production in plants is modulated by various types of stress. Each compound and its corresponding plant response provide valuable insights into the adaptive strategies plants use to manage environmental and biotic challenges. For instance, in *Cajanus platycarpus*, herbivory by *Helicoverpa armigera* leads to an increase in flavonoid content, suggesting that flavonoids play a defensive role against herbivore damage (Tyagi et al. 2022)**.** Stringlis et al., (2019) highlight that fungal infection by *Microcyclus ulei* in *Hevea brasiliensis* leads to increased levels of scopoletin, indicating its role in fungal pathogen defense. This finding is consistent with observations in *Plantanus occidentalis*, which also shows elevated levels of both coumarin and scopoletin in response to fungal infections. These results highlight the importance of these compounds in the plant’s defense mechanisms against fungal pathogens. Moreover, structural defenses are prominently observed in response to pathogen stress. Increased lignin production in *Pinus nigra* and *Triticum* spp., due to blight disease and stem rust respectively, suggests that lignification serves as a common mechanism to enhance structural defense against pathogens (Moura et al. 2010).

In addition to structural adaptations, phenolic acids also play a crucial role in plant defense. *Zea mays* increases caffeic acid esters in response to leaf blight, highlighting the protective role of these phenolic compounds (Pusztahelyi et al. 2016). Similarly, *Vigna radiata* responds to nematode infection by elevating chlorogenic and trans-cinnamic acids, which contribute to its defense mechanisms (Ahmed et al. 2009). Rutin is another compound that enhances plant immune responses. In *rice*, *tobacco*, and *Arabidopsis thaliana*, rutin boosts resistance to bacterial infections and primes the expression of pathogenesis-related genes, underscoring its role in plant immunity (Yang et al. 2016).

The impact of stress on SM production is also evident in responses to fungal pathogens. For example, exposure to Fusarium wilt increases cinnamic acid levels in cucumber, which, while contributing to defense, also induces oxidative stress and stimulates antioxidant enzyme activities as a protective response (Ye et al. 2006). Similarly, naringenin production is enhanced in tobacco in response to *Phytophthora nicotianae* infection, contributing to antimicrobial activity and increased pathogen resistance (Sun et al. 2022). Ginsenosides, produced by *Panax* species such as *Panax ginseng* and *Panax quinquefolius*, also increase in response to fungal infections, suggesting their role in both defense and adaptation (Biswas et al. 2016; Hao et al. 2020)***.*** Furthermore, diterpenes and monoterpenes are involved in antimicrobial defense. *Ginkgo biloba* and *Mentha spicata* produce ginkgolides and menthol, respectively, in response to pathogen stress, illustrating the defensive role of these compounds (Jung et al. 2003; Lin et al. 2017). Lastly, the broad role of flavonoids in plant defense is highlighted by their increased production in response to various stresses, including pathogens and herbivores. This reflects their significant contribution to plant defense mechanisms (Hou et al. 2004; Jain et al. 2015).

**Fig. 2:**
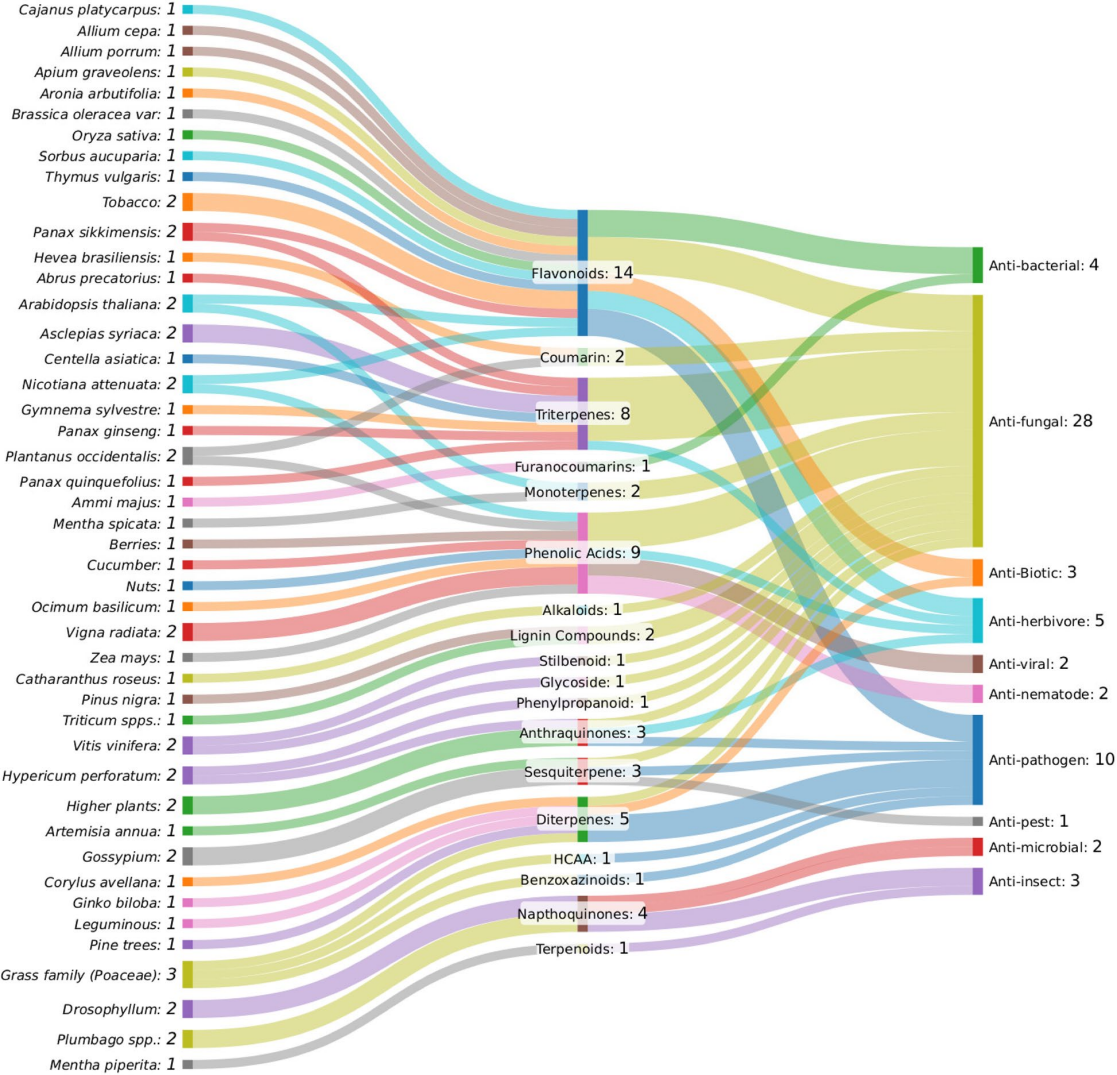
Sankey Diagram represents the intricate connections between plant species, secondary metabolites, and biotic stress responses. The diagram was created with SankeyMATIC

In general, as illustrated in Fig. 2, three predominant classes of secondary metabolites—flavonoids (14 links), phenolic acids (9 links), and triterpenes (8 links)—are primarily associated with biotic stress tolerance, particularly in antifungal and antibacterial defense mechanisms.

## Functional genes of plant SMs

Plants have evolved sophisticated and complicated defensive mechanisms against both biotic and abiotic stresses. Transcription factors (TFs) can regulate downstream gene expression via a complex network and play a critical role in the plant stress response. There are now 58 transcription factor families identified in plants, including WRKY, MYB (Myeloblastosis), bHLH,( Basic Helix-Loop-Helix) BZIP (Basic Leucine Zipper), NAC (NAM, ATAF, and CUC), and AP2/ERF (APETALA2/Ethylene Responsive Factor) (Table 1).

**Table 1:** Plant TFs mediate abiotic and biotic stress response by regulating SM biosynthesis

TF families	Transcriptional factors	Plant species	Secondary metabolites	Resistance/pathogen	References
AP2/ERF	JRE4 (GAME9)	*Solanum tuberosum*	SGAs	*Spodoptera litura*	(Cárdenas et al. 2016)
	NtERF32	*Nicotiana tabaccum*	Nicotine	Defence against Herbivory	(Sears et al. 2014)
	ORA59	*Arabidopsis thaliana*	HCAA	*Alternaria brassicicola,* *Botrytis cineria*	(Li et al. 2018)
	EREB58	*Zea mays*	Sesquiterpenes	Defence against herbivory	(Li et al. 2015a)
	GbERF1	*Gossypium barbadense*	Lignin	*Verticillium dahliae*	(Guo et al. 2016)
	TcERF12/15	*Taxus chinensis*	Taxol	*Phytophthora capsici*	(Zhang et al. 2015)
	VqERF114	*Vitis quinquangularis*	Resveratrol	*Botrytis cinerea*	(Wang and Wang 2019)
	PnERF1	*Panax notoginseng*	Saponins	*Fusarium monilliforme*	(Deng et al. 2017)
	TaAP2–15	*Triticum aestivum*	Terpenoids	*Puccinia striiformis*	(Hawku et al. 2021)
	ScAPD1	*Syntrichia caninervis*	Lignin	*Verticillium dahliae*	(Li et al. 2023)
	OsERF71	*Oryza sativa*	Lignin	*Drought stress*	(Lee et al. 2017)
WRKY	StWRKY1	*Solanum tuberosum*	HCAAs	*Phytophthora infestans*	(Yogendra et al. 2015)
	TaWRKY70	*Triticum aestivum*	HCAAs	*Fusarium graminearum*	(Kage et al. 2017)
	HvWRKY23	*Hordeum vulgare*	HCAAs	*Fusarium graminearum*	(Karre et al. 2019)
	StWRKY8	*Solanum tuberosum*	BIAs	*Phytophthora infestance*	(Yogendra et al. 2017)
	ZmWRKY79	*Zea mays*	Terpenoid phytoalexins	*Rhizoctonia solani*	(Fu et al. 2018)
	SsWRKY18/ 40	*Saliva sclarea*	Diterpenoids	Anti-microbial antifungal	(Alfieri et al. 2018)
	VvWRKY1	*Vitis vinifera*	Jasmonic acid	*Plasmopara viticola*	(Marchive et al. 2013)
	Na WRKY3/6	*Nicotiana attenuata*	Volatile terpenes	*Manduca sexta*	(Skibbe et al. 2008)
	TcWRKY1	*Tillia chinenesis*	Taxol	Anti-microbial	(Li et al. 2013)
	WsWRKY1	*Withania somnifera*	Triterpenoid	*Pseudomonas syringae*, *Botrytis cinerea, Spodoptera litura*	(Singh et al. 2017)
	VvWRKY24/03/8	*Vitis vinifera*	Resveratrol	*Botrytis cineria*	(Vannozzi et al. 2018; Jiang et al. 2019)
	GhMKK2/GhNTF6	*Gossypium hirsutum*	Flavonoids	*F. oxysporum* f. sp. *vasinfectum*	(Wang et al. 2022)
	ZmWRKY83	*Zea mays*	Flavonoids and Terpenoid	*Fusarium graminearum*	(Bai et al. 2021)
	GmWRKY136/53/86	*Glycine max*	Salicylic acid	*soybean cyst nematode Heterodera glycines*	(Yang et al. 2017)
	SlWRKY45/3/70	*Solanum lycopersicum*	Phenolics	*meloidogyne javanica*	(Chinnapandi et al. 2017, 2019)
	OsWRKY45 /46	*Oryza sativa*	Ethylene, H2O2	*Bemisia tabaci,*	(Huang et al. 2016)
	GhWRKY41	*Gossypium hirsutum*	Lignin and Flavonoids	*Verticillium dahliae*	(Xiao et al. 2023)
bHLH	ILR3/bHLH104, bHLH04/05/06	*Arabidopsis thaliana*	GLs	*Heterodera schachtii*	(Frerigmann et al. 2014; Samira et al. 2018)
	VvbHLH1	*Arabidopsis thaliana*	Flavonoids	Drought and salt stress	(Wang et al. 2016a)
	MdMYC2	*Malus domestica*	Anthocyanin	Anti-pathogenic, drought and salt stress	(An et al. 2016)
	TSAR1/TSAR2	*Medicago falcata*	Saponins	Anti-microbial	(Mertens et al. 2016)
	DPF	*Oryza sativa*	Diterpenoid phytoalexins	*Magnaporthe grisea*	(Yamamura et al. 2015)
	LjbHLH7	*Miyakogusa*	Cyanogenic glucosides	*Phytophthora palmivora*	(Chen et al. 2022)
	PalbHLH1	*Populus alba*	Flavonoids	*Dothiorella gregaria, Botrytis cinerea*	(Bai et al. 2020)
	SlbHLH22	*Solanum lycopersicum*	Flavonoids	Drought and salt stress	(Waseem et al. 2019)
	AmDEL	*Arabidopsis thaliana*	Flavonoids	Drought and salt stress	(Wang et al. 2016c)
bZIP	MdHY5	*Malus domestica*	Anthocyanin	Light stress	(An et al. 2016)
	AtHY5	*Arabidopsis thaliana*	Anthocyanin	Light stress	(Shin et al. 2013)
	SlHY5	*Solanum lycopersicum*	Anthocyanin	Light stress	(Liu et al. 2018)
	OsTGAP1	*Oryza sativa*	Diterpenoid phytoalexins	Anti-pathogenic	(Miyamoto et al. 2014; Yoshida et al. 2017)
	OsbZIP79	*Oryza sativa*	Diterpenoid Phytoalexins	*Xanthomonas oryzae*	(Miyamoto et al. 2015)
	CAbZIP1	*Capsicum annuum*	Flavonoids	*Xanthomonas campestris*	(Lee et al. 2006)
MYB	AtMYB34/51/122	*Arabidopsis thaliana*	IGS	*Plectospharella cucumerina*	(Frerigmann and Gigolashvili 2014; Frerigmann et al. 2016)
	AtMYB75	*Arabidopsis thaliana*	Anthocyanin	*Pieris brassicae*	(Onkokesung et al. 2014)
	VvMYBC2-L1	*Vitis vinifera*	Proanthocyanidins	Wounding and Oxidative stress	(Huang et al. 2014)
	RrMYB5/10	*Rosa rugosa*	Proanthocyanidins	Wounding and Oxidative stress	(Shen et al. 2019)
	AtMYB11/111	*Arabidopsis thaliana*	Flavonoids	Drought	(Wang et al. 2016b)
	AtMYB12	*Arabidopsis thaliana*	Flavonoids	*Spodoptera litura, Helicoverpa armigera*	(Misra et al. 2010)
	AtMYB12	*Arabidopsis thaliana*	Flavonoids	*Heterodera schachtii, Meloidogyne incognita*	(Hamamouch et al. 2020)
	SbMYB8	*Scutellaria baicalensis*	Flavonoids	Drought	(Yuan et al. 2015)
	CsMYB2/26	*Camellia sinensis*	Flavonoids	*Exobasidium vexans*	(Wang et al. 2018a)
	OsMYB30/55/110	*Oryza sativa*	HCAA	*Xanthomonas oryzae*	(Kishi-Kaboshi et al. 2018)
	CsMYB1	*Citrus sinensis*	Flavonoids and HCAA	*Elsinoe fawcettii*	(Liu et al. 2016)
	CsMYB96	*Citrus sinensis*	Lignin	*Penicillium italicum*	(Zhang et al. 2021)
	GhODO1	*Gossypium hirsutum*	Lignin	*Verticillium dahliae*	(Zhu et al. 2022)
	PtMYB115	*Populus trichocarpa*	Proanthocyanidins	*Dothiorella gregaria*	(Wang et al. 2017)
	AtMYB96	*Arabidopsis thaliana*	Salicylic acid	*Pseudomonas syringae, drought*	(Seo and Park 2010)
	TaMyb1D	*Nicotiana tabacuma*	Flavonoids	*drought and oxidative stress*	(Wei et al. 2017)
NAC	PtrNAC72	*Poncirus trifoliata*	Putriscene	Drought stress	(Wu et al. 2016)
	PaNAC03	*Picea abies*	Flavonoids	*Heterobasidion annosum*	(Dalman et al. 2017)
	ANACO32	*Arabidopsis thaliana*	Anthocyanin	Drought and salt stress	(Mahmood et al. 2016)
	HbNAC1	*Hevea brasiliensis*	Latex	Drought stress	(Cao et al. 2017)
	MfNACsa	*Medicago falcata*	Glutathione	Drought stress	(Duan et al. 2017)
	ANAC042	*Arabidopsis*	Camalexin	*Alternaria brassicicola*	(Saga et al. 2012)
	MdNAC52	*Malus domestica*	Anthocyanin and proanthocyanidins	*Xanthomonas campestris*	(Sun et al. 2019)
	TaNAC032	*Triticum aestivum*	Lignin	*Fusarium graminearum*	(Soni et al. 2021)
	SlNAP1	*Solanum lycopersicum*	Terpenoids	*Ralstonia solanacearum*	(Wang et al. 2020)
	OsNAC9/OsTF1L	*Oryza sativa*	Lignin	*Drought stess*	(Redillas et al. 2012)

*SGAs* Steroidal glycoalkaloids, *HCAA*, *GLs*, *IGS* Indolic glucosinolate

In the context of regulating enzyme genes, TFs possess the ability to integrate both external and internal signals, allowing them to control the SMs accumulation. Consequently, the expression of genes in connection with the biosynthetic pathway of SMs is intricately influenced by multiple TFs operating at various regulatory levels (Yang et al. 2012).

### WRKY TFs

The WRKY family is one of the largest families of transcriptional regulators, widely present in plant genomes. These proteins play a crucial role in plant growth, development, SM synthesis, and tolerance to both biotic and abiotic stresses (Rushton et al. 2010).

The WRKY proteins possess a highly conserved domain, termed the WRKY domain, consisting of approximately 60 amino acid residues (Eulgem et al. 2000). A key characteristic of these proteins is the presence of the WRKYGQK sequence at the N-terminal, alongside a C2H2 or C2HC zinc finger motif at the C-terminus. Based on the variations in WRKY domains and the type of zinc finger motifs, the WRKY family is categorized into three major groups: Group I, Group II, and Group III. Group I members contain two WRKY domains and a C2H2-type zinc finger, while both Group II and Group III members possess a single WRKY domain with C2H2 and C2HC zinc finger motifs, respectively (Bakshi and Oelmüller 2014; Rinerson et al. 2015). Additionally, Group II proteins are further subdivided into five primary subgroups (IIa, IIb, IIc, IId, and IIe) based on the evolutionary relationships among their WRKY domains (Rushton et al. 2008; Chen et al. 2023; Ma et al. 2024). With the increasing availability of sequenced genomes, the identification and characterization of the WRKY gene family have been extensively studied in both model and crop species (Wang et al. 2011; Baillo et al. 2020; Mu et al. 2023; Tang et al. 2023). In addition to their involvement in stress responses, WRKY TFs also play a critical role in the production of SMs by modulating the expression of genes involved in the biosynthesis of compounds such as phenylalanines, alkaloids, terpenoids, and their subclasses (Phukan et al. 2016; Godbole et al. 2022; Ren et al. 2023; Wang et al. 2023).

Numerous studies have demonstrated that WRKY TFs modulate SM biosynthesis in plants, which can be harnessed to enhance resistance against pathogens and abiotic stressors. For instance, in *Solanum tuberosum*, StWRKY8 enhances resistance to late blight caused by *Phytophthora infestans* by upregulating the expression of the *TyDC*, *NCS*, and *COR2* genes, which are key components of the biosynthetic pathway responsible for producing benzylisoquinoline alkaloids (Yogendra et al. 2017).

Similarly, VvWRKY24 and VvWRKY3 play pivotal roles in the biosynthesis of resveratrol in *Vitis vinifera* by modulating of the Stilbene synthase (STS) pathway, which confers antimicrobial resistance against *Botrytis cineria* (Vannozzi et al. 2018). Marchive et al., (2013) demonstrated that VvWRKY1 enhances resistance to downy mildew in *Vitis vinifera* through transcriptional reprogramming that activates the jasmonic acid signaling pathway. Moreover, VvWRKY8 in grapevine regulates resveratrol biosynthesis by repressing *STS* genes via direct interaction with VvMYB14 (Jiang et al. 2019).

Several studies have also shown that various WRKY genes are regulate hydroxycinnamic acid amide (HCAA) biosynthesis through modulation of the phenylpropanoid pathway. For example, SlWRKY1 confers resistance to late blight caused by *Phytophthora infestans* (Yogendra et al. 2015). In wheat and barley, TaWRKY70 and HvWRKY23 acts as positive regulators of resistance against *Fusarium graminearum* by activating genes associated with HCAA biosynthesis (Kage et al. 2017; Karre et al. 2019). Furthermore, in native tobacco (*Nicotiana attenuate*), WRKY3 and WRKY6, are involved in the the biosynthesis of volatile terpenes and exhibit strongly upregulation after attack by *Manduca sexta* (Skibbe et al. 2008). In maize, ZmWRKY79 positively regulates phytoalexin biosynthetic genes, and its overexpression in tobacco confers resistance against *Rhizoctonia solani* infection (Fu et al. 2018).

WRKY TFs also play a significant role in plant resistance against pathogenic microorganisms via the MAPK pathway. In cotton, GhWRKY41 activates the expression of *GhC4H* and *Gh4CL*, promoting the accumulation of lignin and flavonoids, thereby enhancing resistance against *Verticillium dahliae* (Xiao et al. 2023). Similarly, Wang et al., (2022) revealed that group IIc WRKY TFs regulate *Fusarium oxysporum*-inducted flavonoid biosynthesis by activating the GhMKK2-mediated pathway. In maize, ZmWRKY83 regulates resistance to gibberella stalk rot caused by *Fusarium graminearum* (Bai et al. 2021). In *Salvia sclarea*, SsWRKY18 and SsWRKY40 regulate the production of abietane-type diterpenoids, with overexpression lines accumulating levels of these compounds, conferring resistance against various bacterial and fungal species (Alfieri et al. 2018).

WRKY TFs also enhance plant defenses against nematodes. For instance, overexpression of GmWRKY 136, 53, and 86 in *Glycine max* enhances resistance to cyst nematodes (Yang et al. 2017). However, during root-knot nematode (*Meloidogyne javanica*) infection of tomato roots, overexpression of SlWRKY45 and SlWRKY3 increases susceptibility, associated with decreased expression of JA- and SA-marker genes (Chinnapandi et al. 2017, 2019).

Furthermore, TcWRKY1 regulates taxol accumulation in *Taxus chinensis*, a compound known for its antimicrobial properties (Li et al. 2013). In *Withania somnifera*, the production and accumulation of triterpenoid withanolides, derived from the phytosterol pathway, are regulated by WsWRKY1. Overexpression of WsWRKY1 decreases susceptibility to bacterial growth, fungal infection, and insect feeding (Singh et al. 2017). Huang et al., (2016) reported that WRKY Group III responds to tomato yellow leaf curly virus (TYLCV) in *Solanum lycopersicum*.

### NAC TFs

The NAC (NAM, ATAF1/2, and CUC2) TF family, one of the largest in plants, is critical for various biological processes, including plant growth, development, and responses to both abiotic and biotic stresses (Han et al. 2023). NAC TFs contain a conserved DNA-binding domain at their N-terminus and an activation domain in their C-terminus. Although many NAC TFs have been identified as key regulators in stress responses, the molecular mechanisms by which they mediate stress responses through secondary metabolism are less well understood, with only a few cases reported.

For instance, in response to drought stress, PtrNAC72 from *Poncirus trifoliate* regulates putrescine biosynthesis by controlling the expression of the *arginine decarboxylase* (*ADC*) gene, leading to increased accumulation of reactive oxygen species (Wu et al. 2016). Glutathione, a key antioxidant metabolite, neutralizes the harmful effects of drought-induced reactive oxygen species (Pompella et al. 2003). In *Medicago falcate,* MfNACsa acts as transcriptional regulator of the gene expression of *glyoxalase1* (GLO1), helping to maintain glutathione levels and enhancing drought tolerance (Duan et al. 2017).

In Arabidopsis, the biosynthesis of anthocyanin is negatively regulated by ANACO32. Overexpression of ANACO32 leads to the downregulation of the *DFR*, *ANS*, and *LODX* genes, which are essential for anthocyanin biosynthesis under stress conditions (Mahmood et al. 2016). The NAC family member HbNAC1, identified in *H. brasiliensis*, is involved in latex biosynthesis and drought tolerance, binding to the CACG motif in the of promoter site of small rubber particle protein (SRPP) to regulate latex biosynthesis (Cao et al. 2017).

NAC TFs have also been shown to enhance tolerance against microbial infections through SM accumulation. In apple, the *MdNAC52* gene is potent regulators of anthocyanin and proanthocyanidins biosynthesis in response to *Xanthomonas* Leaf spot (Sun et al. 2019). In Norway spruce, the NAC gene PaNAC03 negatively regulates flavonoid biosynthesis genes, like *CHS*, *F3’H*, and *LAR3*, increasing resistance against *Heterobasidion annosum* (Dalman et al. 2017).

In Arabidopsis, ANAC042 has been characterized as a regulator of camalexin biosynthesis, increasing tolerance against *Alternaria brassicicola* infection (Saga et al. 2012). Similarly, TaNAC032, OsNAC9, and OsTF1L regulate lignin biosynthesis genes, promoting resistance to *Fusarium* head blight in wheat and drought in rice (Redillas et al. 2012; Soni et al. 2021). The SlNAP gene in tomato, identified by Wang et al. (2020), significantly enhances defense against *Ralstonia solanacearum* when overexpressed (Wang et al. 2020).

### bZIP TFs

The basic leucine zipper (bZIP) TFs are ubiquitous in plants and play a pivotal role in regulating development and stress responses. These TFs characterized by a highly conserved bZIP domain, which includes a region rich in basic amino acids and a leucine zipper (Wang et al. 2024). Numerous studies have identified the regulation of SMs by bZIP proteins, highlighting their involvement in plant stress responses. Specifically, bZIP TFs have been shown to be crucial in anthocyanin biosynthesis across various plant species. For instance, *HY5* genes (*AtHY5*. *MdHY5*, and *SlHY5*) positively regulate anthocyanin accumulation in response to light in *Arabidopsis thaliana* (Shin et al. 2013), *Malus domestica* (An et al. 2017), and *Solanum lycopersicum* (Lee et al. 2006). Moreover, bZIP TFs also influence the biosynthesis of flavonoid and terpenoid phytoalexins, which are key pathogen-resistant compounds in rice (Miyamoto et al. 2014, 2015; Yoshida et al. 2017).

In *Oryza sativa*, OsTGAP1 positively regulates the activation of OsDXS3, thereby contributing to the production of momilactones—specialized antimicrobial metabolites—by directly controlling OsDXS3 expression (Miyamoto et al. 2014). Conversely, overexpression of *OsbZIP79* resulted in the downregulation of phytoalexin biosynthetic genes and specific genes MEP pathway, such as *DXS*, leading to reduced phytoalexin accumulation (Miyamoto et al. 2015).

### MYB TFs

The MYB TFs represent the largest class of transcriptional regulators in plants, with pivotal roles in development, metabolism, and responses to both abiotic and biotic stresses. These TFs typically contain one or more conserved structural domains, each comprising 51 or 52 amino acids arranged in a helix-turn-helix spatial pattern. The *MYB* gene family is classified into four subfamilies: 1R-MYB (MYB-related), 2R-MYB (R2R3-MYB), 3R-MYB (R1R2R3-MYB), and 4R-MYB (R1R2R2R1/2-MYB), with 2R-MYB TFs being the most abundant in plants (Dubos et al. 2010). MYB TFs are often involved in mediating plant stress responses through the regulation of SMs.

Flavonoids are among the most commonly accumulated compounds regulated by *MYB* genes in many plant species. For instance, in tea plants, flavonoid biosynthesis is controlled by CsMYB2 and CsMYB26, which directly bind to the promoters of *CsF3'H* and *CsLAR*, two key flavonoid biosynthetic genes. These expression of these MYB TFs is inducible and correlates with their target genes in response to pathogen infection (Wang et al. 2018a). Similarly, SbMYB8 in *Scutellaria baicalensis* positively regulates flavonoid biosynthesis by activating the expression of *SbCHS* and enhancing drought tolerance (Yuan et al. 2015). In Triticum aestivum, overexpression of TaMYB1D in tobacco plants enhanced drought and oxidative stress by modulating phenylpropanoid metabolism (Wei et al. 2017).

Seo and Park, (2010) reported that AtMYB96 plays a pivotal role in linking ABA-mediated abiotic stress signaling with salicylic acid biosynthesis, thereby enhancing resistance to both drought stress and infection by the virulent *Pseudomonas syringae* (Seo and Park 2010)*.* Additionally, Zhang et al 2021 demonstrated that the R2R3-MYB TF CsMYB96, a homolog of AtMYB96, promotes the host defense against *Penicillium italicum* in citrus by increasing lignin content via the phenylpropanoid pathway (Zhang et al. 2021). Another citrus MYB factor, MYBF1, has been found to regulate the synthesis of specific flavonoids by activating the expression of the *chalcone synthase* (*CHS*) gene (Liu et al. 2016; Wang et al. 2018b).

In Arabidopsis, flavonols contribute to plant defense against nematodes (*Heterodera schachtii and Meloidogyne incognita*) through regulation by AtMYB12. This MYP TF controls flavonol biosynthesis genes such as *CHS* and *FLS1* (*flavonol synthase*). (Hamamouch et al. 2020). Moreover, AtMYB11, AtMYB12, and AtMYB111 are identified as key regulators of flavonoid biosynthesis. Overexpression of these genes has been shown to increase flavonoid levels in various plant species, including tomato and tobacco (Pandey et al. 2014, 2015; Li et al. 2015b). This increase in flavonoids enhances tolerance to insect pests such as *Spodoptera litura* and *Helicoverpa armigera* (Misra et al. 2010), as well as to abiotic stresses like drought (Wang et al. 2016b).

In poplar trees, overexpression of PtMYB115 results in enhanced resistance to the fungal pathogen *Dothiorella gregaria* throught increased proanthocyanidin content (Wang et al. 2017). Similarly, GhODO1*,* isolated from cotton*,* regulates the key genes *Gh4CL1* and *GhCAD3*, which are important for lignin synthesis, thereby increasing resistance to the fungal pathogen *Verticillium dahlia* (Zhu et al. 2022)*.* In rice, three MYB proteins—MYB30, MYB55, and MYB110—activate phenylpropanoid biosynthetic genes such as *HCT*, *4CL3*, *C4H*, and *PAL*, which regulate HCCAs accumulation and confer resistance against fungal and bacterial pathogens (Kishi-Kaboshi et al. 2018). Triple mutant lines of Arabidopsis—*myb34*, *myb51*, and *myb122*—showed a considerable reduction in indole glucosinolate accumulation, resulting in increased susceptibility to *Plectosphaerella cucumerina* (Frerigmann and Gigolashvili 2014; Frerigmann et al. 2016). Overexpression of AtMYB75 enhanced plant protection against pests caused by *Pieris brassicae* by increasing anthocyanin and flavonol levels (Onkokesung et al. 2014). Shen et al., (2019) and Huang et al., (2014) demonstrated that RrMYB5/MYB10 from *Rosa rugosa* and VvMYBC2-L1 from *Vitis vinifera* respond to wounding and oxidative stresses while enhancing anthocyanin biosynthesis by promoting the expression of *DFR* (dihydroflavonol reductase), ANR (anthocyanidin reductase), and *LAR* (leucoanthocyanidin reductase) genes (Huang et al. 2014; Shen et al. 2019).

### bHLH TFs

The basic helix-loop-helix (bHLH), ubiquitous across eukaryotes, represent the second largest class of plant transcription factors after the MYBs. These bHLH TFs are characterized by a conserved domain of approximately 60 residues, comprising two distinct motifs: a basic region and a helix-loop-helix (HLH) region. They play a vital roles in diverse biological processes, including responses to various stresses in plants (Li et al. 2021). bHLH TFs are involved in the biosynthesis of several SMs, including flavonoids, terpenoid phythoalexins, glucosinolates, and cyanogenic glucosides.

In Arabidopsis, four *bHLH* genes (*ILR3/bHLH104, bHLH04, bHLH 05, bHLH 06*) have been shown tovcontribute to defense responses against *Heterodera schachtii* through glucosinolates biosynthesis (Frerigmann et al. 2014; Samira et al. 2018). Some bHLH TFs also function in both biotic and abiotic stresses. For example, overexpression of MdMYC2 from *Malus domestica* in transgenic Arabidopsis lines enhances tolerance to pathogenic diseases as well as drought and salt tolerance (An et al. 2016). Flavonoids are crucial defensive compounds, particularly in response to drought and salt stress in plants. The overexpression of AmDEL from *Antirrhinum majus* and VvbHLH1 from *Vitis vinifera* in transgenic Arabidopsis increases total flavonoid content and enhances tolerance to salt and drought stress (Wang et al. 2016a, c). Similarly, overexpression of SlbHLH22 in transgenic tomato plants increases both drought and salt stress tolerance while promoting flavonoid accumulation (Waseem et al. 2019). Additionally, PalbHLH1 has been identified as a transcriptional activator of flavonoid biosynthesis, providing resistance against infections by *Botrytis cinerea* and *Dothiorella gregaria* (Bai et al. 2021). LjbHLH7, a transcription factor in *Lotus japonicus*, when overexpressed, leads to the accumulation of cyanogenic glucosides and enhances insect resistance (Chen et al. 2022). In *Medicago truncatula*, the bHLH TFs TSAR1 and TSAR2 regulate triterpene saponin biosynthesis by activating the *HMGR* gene (Mertens et al. 2016).

In rice, the diterpenoid phytoalexin factor (DPF) plays a central role in diterpenoid phytoalexin biosynthesis, providing antifungal activity against *Magnaporthe oryzae* (Yamamura et al. 2015).

### AP2/ERF TFs

The AP2/ERF (Apetala2/Ethylene Response Factor) family of TFs is essential to various plant processes, particularly in mediating responses to biotic and abiotic stresses. These TFs are characterized by the AP2/ERF domain, comprises approximately 60–70 amino acids, and is essential for DNA binding (Licausi et al. 2013; Feng et al. 2020). The AP2/ERF family plays a significant role in regulating the biosynthesis of SMs, which are crucial for plant stress responses (Wasternack and Song 2016).

Lignin, a critical component of the plant cell wall, serves as a defensive compound against various pathogens. Overexpression of *GbERF1-like* from cotton (*Gossypium barbadense*) and *ScAPD1-like* from desert moss (*Syntrichia caninervis*) in Arabidopsis significantly enhanced the transcription of lignin biosynthesis genes (*PAL*, *C4H*, *C3H*, *HCT*, *CoMT*, *CCR*, and *F5H*), resulting in increased lignin accumulation and elevated resistance to *Verticillium dahliae* (Guo et al. 2016; Li et al. 2023). Additionally, overexpression of OsERF71 in transgenic rice plants modified root architecture and conferred drought tolerance by upregulating lignin biosynthesis pathway genes (Lee et al. 2017).

HCAAs, a class of antimicrobial metabolites, are involved in plant defense against pathogens. Overexpression of *ORA59* in Arabidopsis, which regulates HCAA biosynthesis, increased resistance to fungal pathogens such as *Botrytis cinerea* and *Alternaria brassicicola* (Li et al. 2018). Similarly, resveratrol, a defense compound against bacterial pathogens, is synthesized by stilbene synthase (STS). In *Vitis quinquangularis*, VqERF114 was found to regulate stilbene biosynthesis by binding to the STS promoter in conjunction with VqMYB35 (Wang and Wang 2019). AP2/ERF TFs also play a essential role in the synthesis of triterpene saponins. In *Panax notoginseng*, overexpression of PnERF1 significantly increased total saponin content (Deng et al. 2017). A study by Li and co-workers (2015) highlighted the involvement of the ERF member EREB58 in regulating sesquiterpene biosynthesis. EREB58 was identified as a master regulator that interacts with the GCC-box motif in the promoter of the sesquiterpene biosynthesis gene *ZmTPS10*, activating its expression. This activation leads to enhanced sesquiterpene production and improved disease resistance in maize (Li et al. 2015a). A recent study in wheat identified TaPR1, a member of the AP2 family, as a positive regulator of resistance to stripe rust fungus (*Puccinia striiformis* f. sp. *Tritici*) (Hawku et al. 2021). In tobacco, NtERF32 specifically binds to the promoter of NtPMT1a, activating its expression and resulting in increased accumulation of nicotine and total alkaloids (Sears et al. 2014). The AP2/ERF family may also regulate bisindole alkaloid biosynthesis. GAME9 (GLYCOALKALOID METABOLISM 9), also known as JRE4 (jasmonate-responsive ERF4), enhances the biosynthesis of steroidal glycoalkaloids (SGAs) in tomato, providing protection against *Spodoptera litura* (Cárdenas et al. 2016; Nakayasu et al. 2017).

**Fig. 3:**
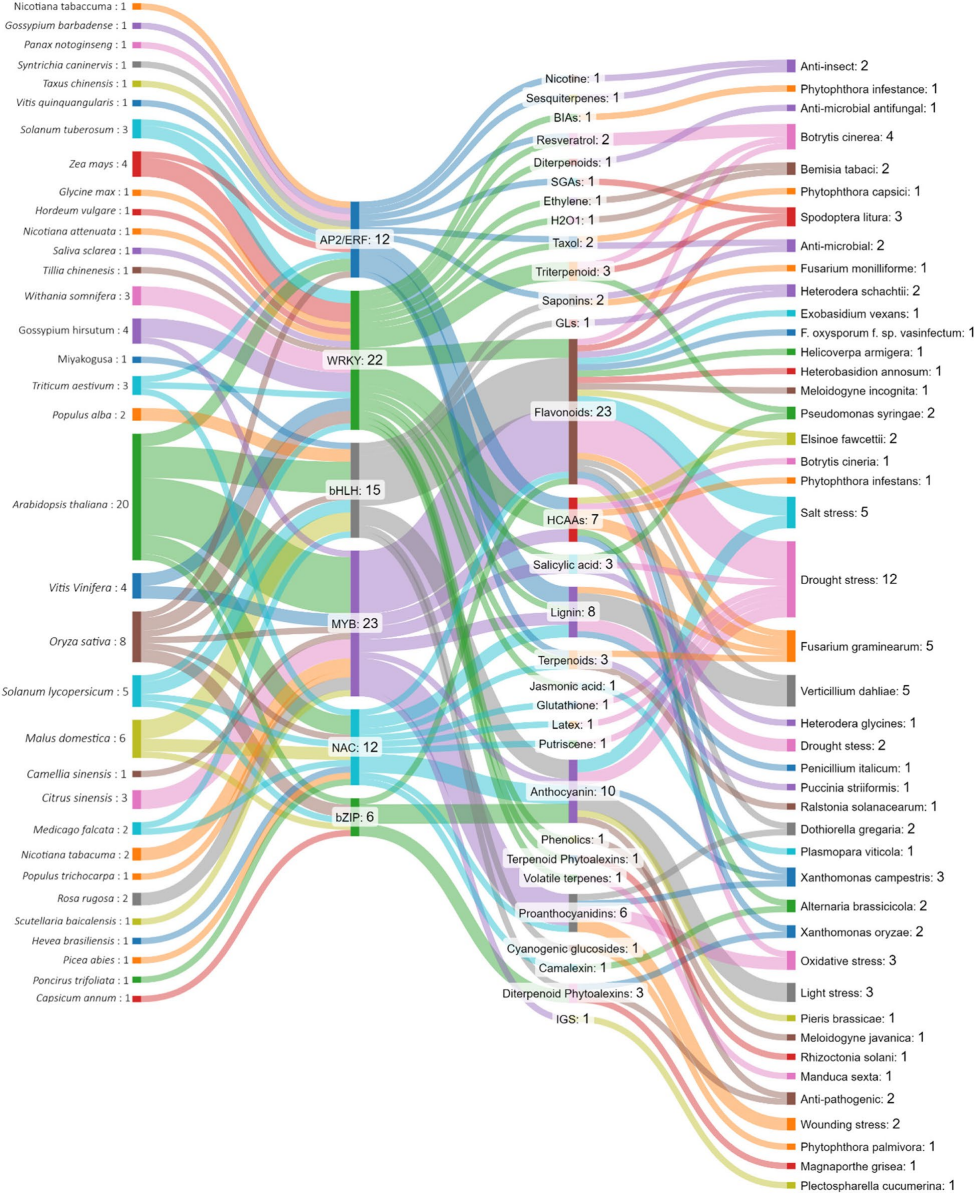
Sankey Diagram illustrates the relationship between plant species, TFs families, SMs and tolerance to abiotic/biotic stresses. The diagram was created with SankeyMATIC

On the whole, the Sankey diagram (Fig. 3) illustres the complex relationships among key TFs, the SMs they regulate, and the specific biotic and abiotic stressors that these metabolites mitigate in plants. The diagram emphasizes the roles of five central TFs—AP2/ERF, WRKY, bHLH, MYB, and NAC—in governing the biosynthesis of various SMs, which enhance plant resilience to environmental stressors.

Among the TFs, WRKY (22 connections) and MYB (23 connections) stand out for their extensive regulatory influence over a broad spectrum of SMs, including flavonoids, phenolics, lignin, and terpenoids. These TFs play pivotal roles in the plant's defense mechanisms against stressors such as drought, pathogens like *Fusarium graminearum* and *Verticillium dahliae*, and oxidative stress. More importantly, flavonoids (23 connections) and phenolic compounds (HCAAs with 8 connections and lignin with 7 connections) emerge as the most frequently synthesized SMs across different plant species, crucial for protection against drought, salt stress, and pathogenic threats.

## Conclusions and future perspectives

**Fig. 4:**
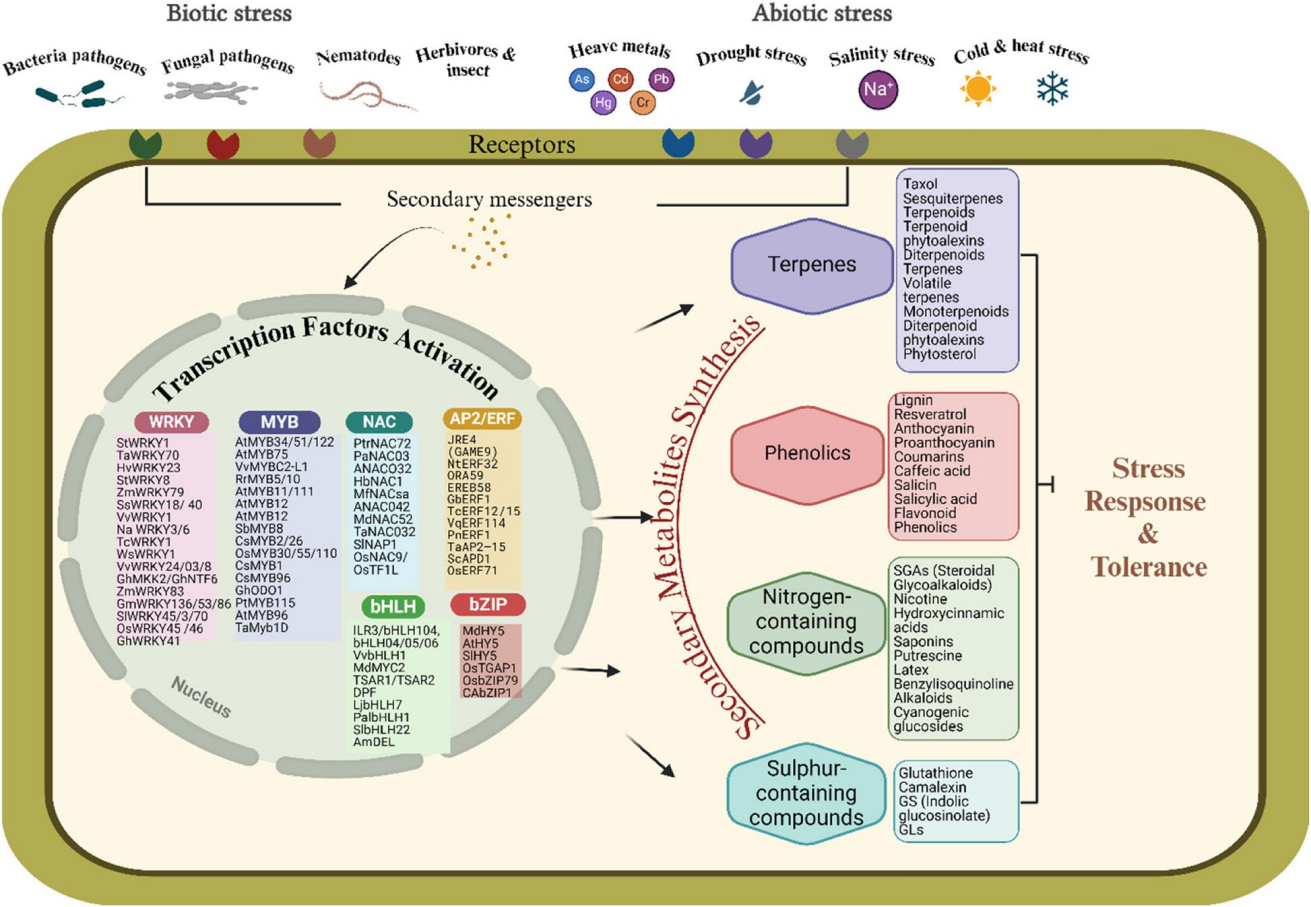
Plants, in their natural environments, are exposed to a wide range of biotic and abiotic stresses, triggering stress defense responses mediated by secondary metabolites. These metabolites are regulated by key transcription factors, including WRKY, MYB, NAC, AP2/ERF, bHLH, and bZIP. The figure was created by Biorender

The complex signaling pathways in plants play a pivotal role in their response to biotic and abiotic stresses. Secondary metabolites, which are natural defense tools utilized by plants, are central to these signaling processes, acting as critical signaling agents and contributing to defense mechanisms and adaptation to adverse environmental conditions. This review provides an overview of the role of secondary metabolites in mediating responses to biotic and abiotic stress through the regulation of transcription factors and genes involved in environmental stress tolerance (Fig. 4). The insights presented in this review can serve as a foundation for further research aimed at identifying key metabolites and transcription factors that could be harnessed to develop stress-tolerant plant varieties.

Future research should prioritize the application of metabolomics and advanced technologies to discover and quantify novel metabolites that may enhance plant resilience to a variety of stressors. Additionally, integrating omics approaches—such as genomics, transcriptomics, proteomics, and metabolomics— combined with bioinformatics will provide a comprehensive understanding of the molecular mechanisms underlying plant stress responses. This holistic strategy will be instrumental in identifying key regulatory genes and metabolites involved in stress signaling pathways.

Under multifactorial stress conditions, plant molecular responses are often unique and may involve pathways or metabolites that have not yet been identified (Rivero et al. 2022; Zandalinas et al. 2022). Moreover, biotic stressors can influence plant responses to abiotic stresses, and vice versa (Rivero et al. 2022). Further studies focusing on the identification of metabolites that function under combined biotic and abiotic stress conditions will be crucial for mitigating the impacts of global warming and climate change on crop productivity.

## Supplementary Information

Supplementary Material 1.

Supplementary Material 2. (Berberich et al. 2015; Schlesinger et al. 2019; Tari et al. 2010).

## Data Availability

Not applicable.

## Abbreviations

4CL3: 4-Coumarate-CoA Ligase 3
ADC: Arginine Decarboxylase
ANR: Anthocyanidin Reductase
ANS: Anthocyanidin Synthase
AP2/ERF: APETALA2/Ethylene Responsive Factor
bHLH: Basic Helix-Loop-Helix
BIAs: Benzylisoquinoline Alkaloids
bZIP: Basic Leucine Zipper
C4H: Cinnamate-4-hydroxylase
CHS: Chalcone Synthase
DFR: Dihydroflavonol 4-Reductase
DPF: Diterpenoid Phytoalexin Factor
DXS: 1-Deoxy-D-Xylulose 5-Phosphate Synthase
F3’H: Flavonoid 3'-Hydroxylase
FLS1: Flavonol Synthase 1
GAME9: Glycoalkaloid Metabolism 9
GLO1: Glyoxalase1
HCAA: Hydroxycinnamic Acid Amides
HCT: Hydroxycinnamoyl-CoA
HMGR: Hydroxymethylglutaryl-CoA Reductase
HQT2: Hydroxycinnamoyl-CoA Quinate Hydroxycinnamoyl Transferase 2
HY5: ELONGATED HYPOCOTYL 5
IGS: Indole Glucosinolates
ILR3: IAA-Leucine Resistant 3
JRE4: Jasmonate-Responsive ERF4
LAR: Leucoanthocyanidin Reductase
LODX: Laccase Oxidoreductase
MEP: Methylerythritol Phosphate
MYB: Myeloblastosis
NAC: NAM, ATAF, and CUC
NAP: NAC-Like, Activated by AP3/PI
PAL: Phenylalanine Ammonia-Lyase
PMs: Primary Metabolites
ROS: Reactive Oxygen Species
SAMDC: S-Adenosylmethionine Decarboxylase
SGAs: Steroidal Glycoalkaloids
SMs: Secondary Metabolites
SRPP: Small Rubber Particle Protein
TFs: Transcription Factors
TSAR1: Triterpene Saponin Biosynthesis Activating Regulator
VOCs: Volatile Organic Compounds
