# Supplementary material for: A comprehensive review of transcription factor-mediated regulation of secondary metabolites in plants under environmental stress
Source: Stress Biol. 2025 Feb 24;5(1):15. doi: 10.1007/s44154-024-00201-w (PMC11850680; doi:10.1007/s44154-024-00201-w)
Supplement: Supplementary file 1 — Supplementary Material 1. [file 44154_2024_201_MOESM1_ESM.docx]

Fig S1: Major compound and their structural formulae

| 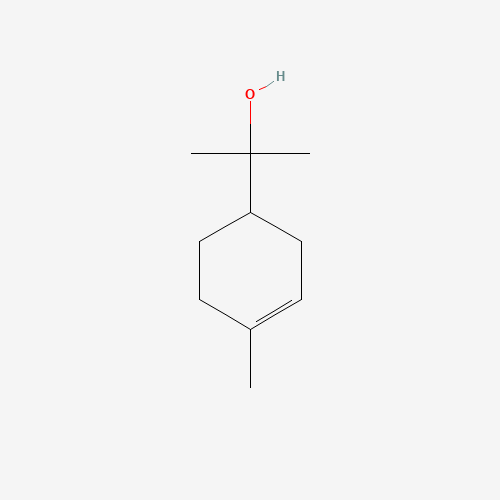  Monotrpenes e.g alpha Terpineol | 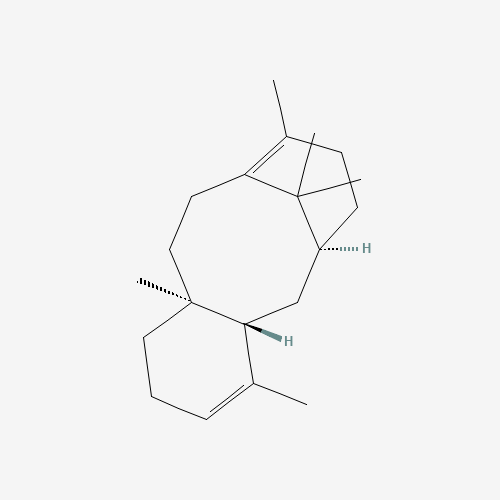  Diterpenes e.g Taxadiene | 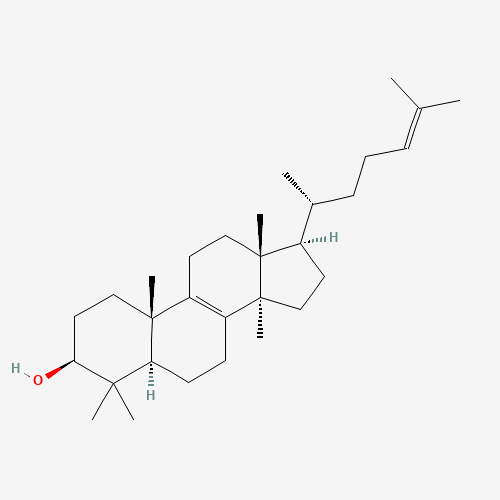  Triterpenes e.g Lanosterol | 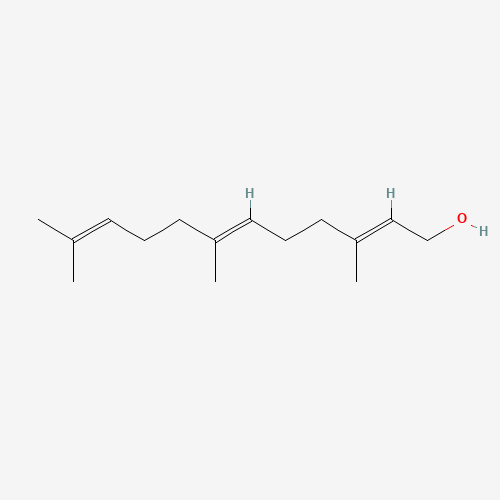  Sesquiterpenes e.g Farnesol |
| --- | --- | --- | --- |
| 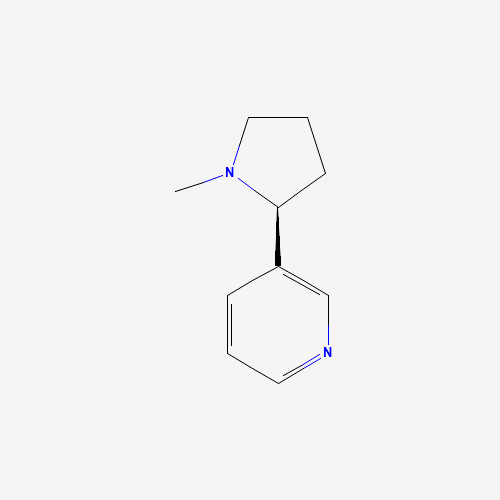  Nicotine | 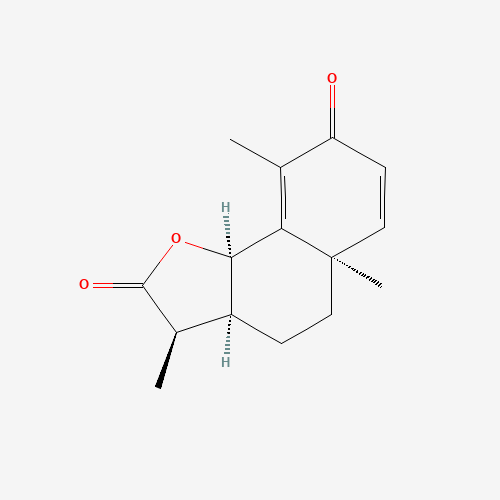  Sesquiterpenes | 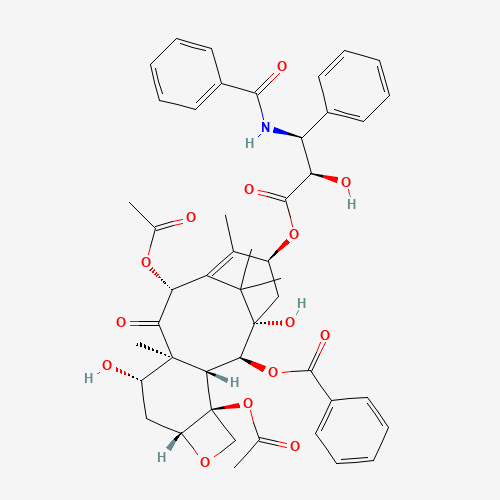  Taxol | 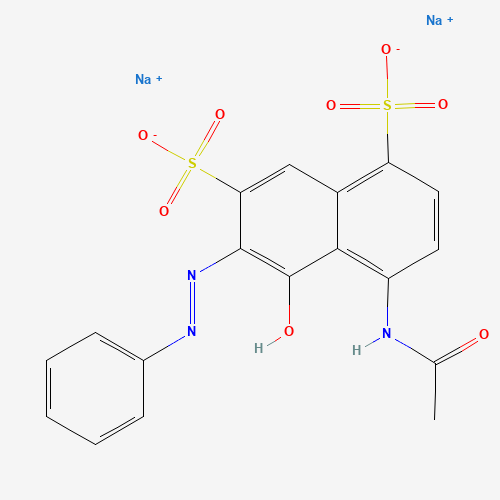  Lignin |
| 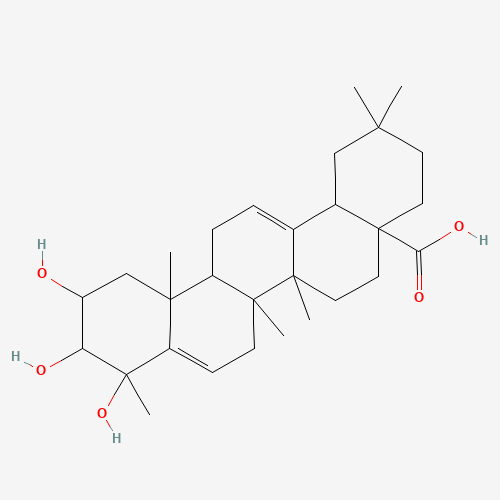  Triterpenoids | 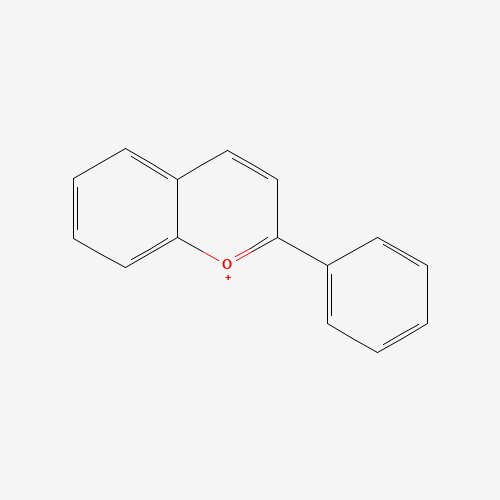  Anthocyanins | 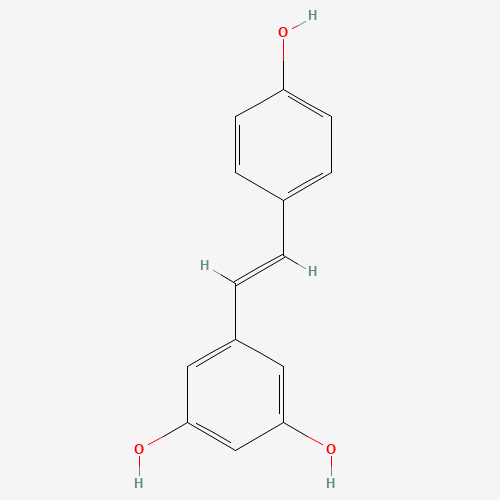  Resveratrol | 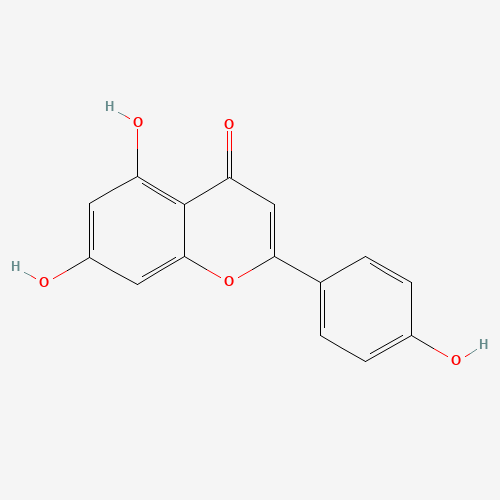  Flavonoids e.g Apigenin |
| 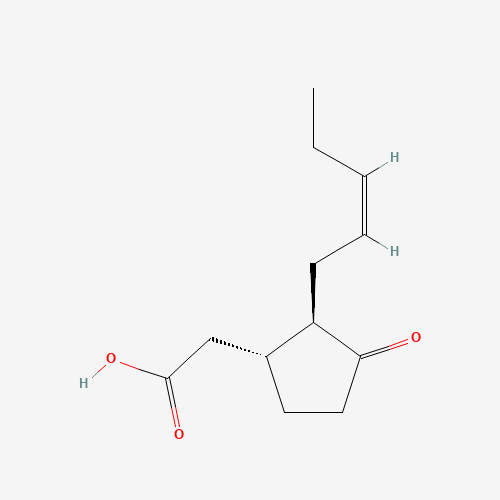  Jasmone acid | 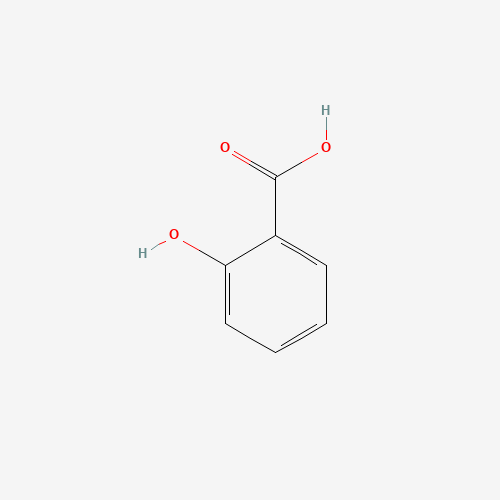  Salicylic acid | 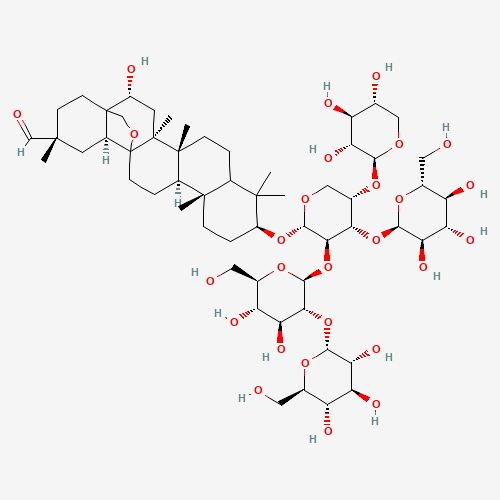  Saponin | 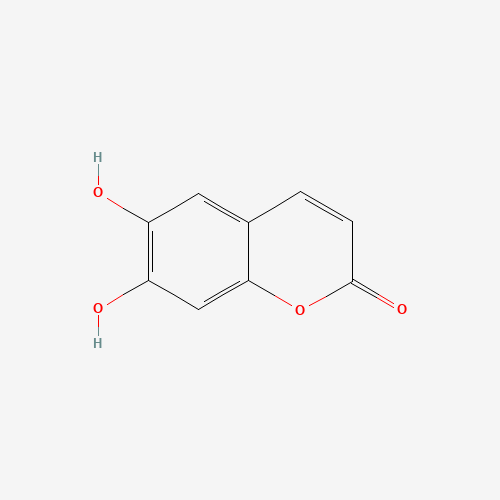  Coumarin e.g Esculetin |
| 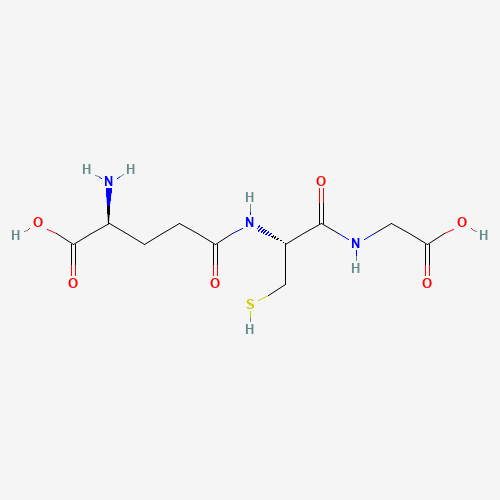  Glutathione | 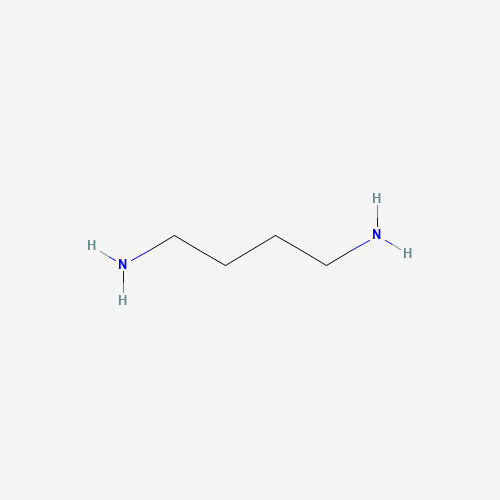  Putrescine | 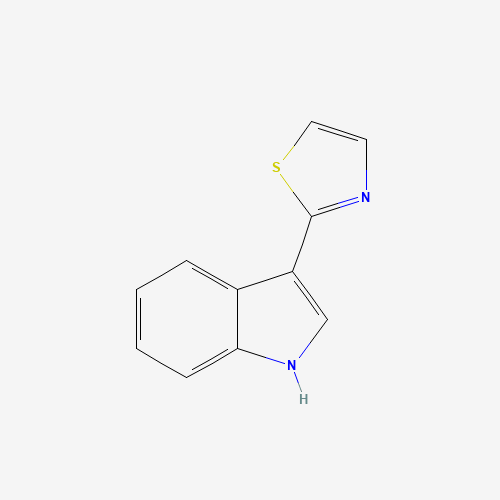  Camalexin | 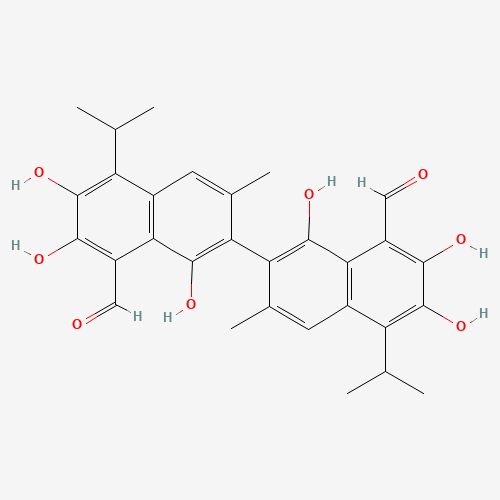  Phytoalexins e.g Gossypol |
