# Supplementary material for: A comprehensive review of transcription factor-mediated regulation of secondary metabolites in plants under environmental stress
Source: Stress Biol. 2025 Feb 24;5(1):15. doi: 10.1007/s44154-024-00201-w (PMC11850680; doi:10.1007/s44154-024-00201-w)
Supplement: Supplementary file 2 — Supplementary Material 2. (Berberich et al. 2015; Schlesinger et al. 2019; Tari et al. 2010). [file 44154_2024_201_MOESM2_ESM.docx]

Table S1. Effect of drought stress on the accumulation of SM in plants

| **Plant species** | **Class**  **of SM** | **SM** | **Effets** | **References** |
| --- | --- | --- | --- | --- |
| *Ocimum basilicum* | Flavonoids | Methyl chavicol and methyleugenol | Increase | (Abdollahi Mandoulakani et al. 2017) |
| *Pennisetum glaucum* |  | NA | Increase | (Ghatak et al. 2022) |
| *Ocimum basilicum* |  | Methyl chavicol and methyleugenol | Increase | (Abdollahi Mandoulakani et al. 2017) |
| *Pisum sativum* |  | MYB | Increase in scavenging activity | (Larson 2017) |
| *Camellia sinensis* |  | Chalcone synthase 1 and 3, Antho-cyanidin reductase 1 and 2 | Increase in catechin accumulation | (Zhang et al. 2016) |
| *Pennisetum glaucum* |  | NA | Increase | (Ghatak et al. 2022) |
| *Salvia dolomitica L.* | Terpenoids | Sesquiterpene | Increase | (Caser et al. 2019) |
| *Vitex agnus-castus* |  | α-pinene, β-terpinyl acetate, caryophyllene, and camphene | Increase | (Rezaei et al. 2019) |
| *Pinus sylvestris* |  | Abietic acid | Increase | (Turtola et al. 2003) |
| *Salvia miltiorrhiza* |  | Tanshinones, cryptotanshinone | Increase | (Turtola et al. 2003) |
| *Salvia dolomitica* |  | Sesquiterpene | Increase | (Caser et al. 2019) |
| *Vitex agnus-castus* |  | α-pinene, β-terpinyl acetate, caryophyllene, and camphene | Increase | (Rezaei et al. 2019) |
| *Lallemantia sp.* | Phenolic compounds | NA | Increase | (Jamal Omidi et al. 2018) |
| *Crataegus laevigata / C. monogyna* |  | Chlorogenic acid , catechin , ( - ) - epicatechin | Increase | (Kirakosyan et al. 2004) |
| *Hypericum brasiliense* |  | Isouliginosin B, rutin , 1,5 - dihydroxyxanthone | Increase | (Nacif de Abreu and Mazzafera 2005) |
| *Salvia miltiorrhiza* |  | Rosmarinic acid | Decrease | (Turtola et al. 2003) |
| *Salvia miltiorrhiza* |  | SmPAL , SmC4H | Decreased shoot and root | (Liu et al. 2011) |
| *Helianthus annuum* |  | HaHQT2 | Increase CQA biosynthesis | (Cheevarungnapakul et al. 2019) |
| *Lallemantia sp.* |  | NA | Increase | (Jamal Omidi et al. 2018) |
| *Salvia miltiorrhiza* |  | Salvianolic acid | Increase | (Turtola et al. 2003) |
| *Vitis vinifera /*  *Chrysanthemum*  *morifolium* |  | Ferulic acids | Increase | (Hodaei et al. 2018) |
| *Glycine max* | Alkaloids | Trigonelline | Increase | (Cho et al. 2003) |
| *Lupinus angustifolius* |  | Chinolizidin | Increase | (Christiansen et al. 1997) |
| *Papaver somniferum* |  | Morphine, codeine | Increase | (Szabó et al. 2005) |
| *Eucomis autumnalis / Scrophularia ningpoensis* | Monoterpenes | Iridoids | Increase | (Masondo et al. 2014) |
| *Mentha spicate / Thymus vulgaris* |  | Cineole | Increase | (Llorens-Molina and Vacas 2017) |
| *Plantago lanceolata* |  | Catalpol | Increase | (Schweiger et al. 2014) |
| *Scrophularia ningpoensis* | Glycosides | _ | Decreased | (Wang et al. 2010) |
| *Scrophularia ningpoensis* |  | Catalpol , harpagide , aucubin , harpagoside. | Increase | (Wang et al., 2010) |

Table S2. Effect of salt stress on the accumulation of SM in plants

| **Plant species** | **Class of SM** | **SM** | **Effets** | **References** |
| --- | --- | --- | --- | --- |
| *Limonium bicolor* | Phenolic compounds, flavonoids | Triethyl citrate, vanillic acid, and epigallocatechin gallate, riethyl citrate, gallic acid, vanillic acid derivative, vanillic acid-hexose, and myricetin-3-O-glucoside/galactoside | Decrease | (Wang et al. 2016) |
| *Ocimum basilicum* |  | Eugenol, methly eugenol | Decrease | (Bahcesular et al. 2020) |
|  | Terpenoids, phenolic acids | Linalool Cichoric and caffeic acids | Increase |  |
| *Mentha spicata* | Terpenoids | Carvone | Increase | (Chrysargyris et al. 2019) |
|  |  | Limonene | Decrease |  |
| *Origanum majorana* | Monoterpenes | Trans-hydrate sabinene Terpinen-4-ol | Decrease | (Baâtour et al. 2011) |
|  |  | Sabinene | Decrease |  |
|  |  | Trans-hydrate sabinene | Increase |  |
| *riganum majorana* |  | Terpinene-4-ol Monoterpene hydrocarbons Phenolic compounds | Increase | (Jelali et al. 2011) |
| *Lycopersicon esculentum* | Sugar alcohol (polyol) | "Sorbitol - 6 - phosphate dehydroge- | Cell structure protection and osmotic adaptation | (Tari et al. 2010) |
| *Oryza sativa* | Polyamines | SAMDC | Increase in polyphenol content | (Berberich et al. 2015) |
| *Triticum aestivum* | Quaternary ammonium compound | Betaine aldehyde dehydrogenase | Reduced membrane permeability lipid peroxidation and H₂O₂ | (Yadav et al. 2017) |
| *Datura innoxia* | Alkaloids | Hyoscyamine 6ẞ - Hydroxylase | Increase of scopolamine | (Schlesinger et al. 2019) |
| *Helianthus annuum* | Phenolic acids | HaHQT2 | Protects against H₂O₂ - induced oxidative stress | (Cheevarungnapakul et al. 2019) |
| *Salvia miltiorrhiza* |  | SmPAL, SmC4H | decreased both shoot and root dry weight | (Liu et al. 2011) |
| *Bixa orellana* | Terpenoids | ẞ - lycopene cyclase | Act as a scavenger | (Sankari et al. 2019) |
| *Daucus carota* |  | Phytoene synthase | Photoprotective functions | (Simpson et al. 2018) |
| *Glycine max* |  | GmbZIP1 | Improved tolerances | (Gao et al. 2011) |

Table S3. Effect of cold and heat stresses on the accumulation of SM in plants

| **Plant species** | **Class**  **of**  **SM** | **SM** | **Effets** | **References** |
| --- | --- | --- | --- | --- |
| ***Cold stress*** | | | | |
| *Cyanea acuminata* | Alkaloids | 10-hydroxy Camptothecin | Increased | (Wang et al. 2003) |
| *Catheranthus Roseus* |  | Vindoline | Increased | (Guo et al. 2007) |
| *Catheranthus Roseus* |  | Catharanthine | Increased | (Guo et al. 2007) |
| *Catharanthus roseus* |  | Vindoline | Decrease | (Dutta et al. 2007) |
| *Quercus Rubra* | Terpenes | Isoprene | Increased | (Hanson and Sharkey 2001) |
| *Brassica Oleracea* |  | a.Quercetin | Increased | (Mølmann et al. 2015) |
| *Daucus Carota* |  | α-farnesene | Increased | (Helmig et al. 2007) |
| *Solanum lycopersicon* | Terpenoids | ẞ - phellandrene, (E)-ß -ocimene | Increase | (Copolovici et al. 2012) |
| *Solanum lycopersicon* |  | 8-elemene, a-humulene and ẞ-caryophyllene (dominant ); in severe cold : ẞ - elemene is produced . | Increase | (Vickers et al. 2009) |
| *Camellia sinensis* |  | nerolidol glucoside | Increase | (Zhao et al. 2020) |
| *Withania somnifera* |  | withanolide A , withaferin A | Increase | (Bilal 2015) |
| *Glycine max* | Phenolics compounds | Genistein , daidzein | Increase | (Janas et al. 2002) |
| *Zea mays* |  | Pelargonidin | Increase | (Christie et al. 1994) |
| *Fagopyrum tartaricum* |  | Anthocyanins (e.g. 3-O-galactosides) and Anthocyanidins (e.g. , malvidin ) | Increase | (Jeon et al. 2018) |
| *Solanum lycopersicon* | Fatty Acyls | (Z)-3-hexenol and (E)-2-hexenal ( dominant ); 1-hexanol and 1,4-hexadienal | Increase | (Vickers et al. 2009) |
| ***Heat stress*** | | | | |
| *Cucumis acuminatus* | Alkaloids | 10 - hydroxycamptothecin | Increase | (Yuan-Gang et al. 2003) |
| *Daucus carota* | Terpenoids | a-erpinolene | Decrease | (Rosenfeld et al. 2002) |
| *Daucus carota* |  | a-caryophyllene , ß - farnesene | Increase | (Rosenfeld et al. 2002) |
| *Quercus rubra* |  | Isoprene (2-methyl-1,3- butadiene) | Increase | (Hanson and Sharkey 2001) |
| *Solanum lycopersicon* |  | Α-humulene | Decrease | (Copolovici et al. 2012) |
| *Centella asiatica* | Phenolics compounds | Asiaticoside | Increase | (Randriamampionona et al. 2007) |
| *Daucus carota* |  | Anthocyanins, coumaric and caffeic acid ; | Increase | (Commisso et al. 2016) |

Table S4. Effect of heavy metals stress on the accumulation of SM in plants

| **Plant** | **Metabolites** | **Risk elements** | **Effect on metabolite** | **Reference** |
| --- | --- | --- | --- | --- |
| *Chrysopogon zizanioides* | Phenolic compounds | As, Cr, Cu, Ni, Pb, and Zn | Increase | (Melato et al. 2012) |
| *Gynura procumbens* | Phenolics, flavonoids, and total saponin content | Cd and Cu | Decrease | (Ibrahim et al. 2017) |
| *Drimia elata* | Total phenolic and flavonoid contents | Cd and Al | Decrease | (Okem et al. 2015) |
| *Phyllanthus amarus Schum.and Thonn* | Phyllanthin and hypophyl- lanthin (Lignans) | Cd | Decrease | (Rai et al. 2005) |
| *Malus domestica , Phaseolus vulgaris ,Triticum aestivum* | Caffeic acid (Phenolics acids) | Zn deficiency | Increase | (Zhang et al. 1991) |
| *Kandelia obovata* | Protocatechuic acid , ferulic acid , and cinnamic acid (Phenolics acids) | Cd and Zn toxicity | Increase | (Chen et al. 2020) |
| *Imperata condensate* | Catechin (Flavonoids) | Cu toxicity | Decrease | (Meier et al. 2012) |
| *Oenothera picensis,*  *Mperata condensate, Lupinus albus, Helianthus annuus* | Cinnamic acid (Phenolic acids) | Cu toxicity | Decrease | (Meier et al. 2012) |
| *Helianthus annuus* | Coumaric acid (Phenolic acids) | Cu toxicity | Increase | (Meier et al. 2012) |
| *Zea mays* | Catechin , catechol , curcumin ,and quercetin (Flavonoids and phenolic compounds) | Al toxicity | Increase | (Kidd et al. 2001) |

Table S5. Effect of UV-B on the accumulation of SM in plants

| **Plant species** | **Class of SM** | **SM** | **Effets** | **References** |
| --- | --- | --- | --- | --- |
| *Catharanthus. roseus* | Alkaloids | Catharanthine | Increased | (Ramani and Chelliah 2007) |
| \|  \| \| --- \|   *Catharanthus roseus* |  | NA | Increased | (Schluttenhofer et al. 2014) |
| *Artemisia annua* L. |  | Artemisinin | Increased | (Mehrotra et al. 2018) |
| *Clarkia breweri* | Phenolic compounds | Eugenol | Increased | (Siddiqui et al. 2009) |
| *Fagopyrum esculentum* |  | Quercetin ; catechin | Increased | (Regvar et al. 2012) |
| *Camptotheca acuminata* |  | NA | Elevate phenolics production | (Takshak and Agrawal 2019) |
| *Gnaphalium luteoalbum* | Flavonoids | Calycopterin | Increased | (Cuadra et al. 1997) |
| *Gossypium viravira* |  | NA | Increased | (Cuadra 2015) |
| *Hordeum vulgare* |  | Caponarin ; luteolin | Increased | (Ra et al. 2020) |
| *Marchantia polymorpha* |  | NA | Increased | (Markham et al. 1998) |
| *Quercus ilex* |  | Kaempferol | Increased | (Skaltsa et al. 1994) |
| \| *Vitis vinifera* \| \| --- \| | Terpenes | NA | Increased tolerance to osmotic stress | (Marchive et al. 2013) |

Table S6: Effect of biotic stress on the accumulation of SM in plants

| **SM Compound** | **Plant Name** | **Type of Stress** | **Response** | **Reference** |
| --- | --- | --- | --- | --- |
| Flavonoid | *Cajanus platycarpus* | *Helicoverpa armigera* | ↑ in flavonoid content | (Tyagi et al. 2022) |
| Scopoletin | *Hevea brasiliensis* | *Microcyclus ulei* | ↑ in scopoletin level | (Stringlis et al. 2019) |
| Coumarin, scopoletin | *Plantanus occidentalis* | *Ceratocystis fimbriata and Ceratocystis platani* | ↑ in coumarin scopoletin level | (Stringlis et al. 2019) |
| Lignin | *Pinus nigra* | *Sphaeropsis sapinea* | ↑ Lignification | (Moura et al. 2010) |
| Lignin | *Triticum spps.* | *Puccinia graminis* | ↑ in lignin level | (Moura et al. 2010) |
| Caffeic acid | *Zea mays* | *Glomerella Graminicola or Cochliobolus heterostrophus* | ↑ in two phenolic caffeic acid esters | (Pusztahelyi et al. 2016) |
| Chlorogenic acid, cinnamic acid | *Vigna radiata* | *Meloidogyne javanica* | ↑ in chlorogenic acid and trans cinnamic acid | (Ahmed et al. 2009) |
| Chlorogenic acid, catechin | *Nicotiana attenuata* | *Trichobaris mucorea* | ↑ in chlorogenic acid and catechin | (Lee et al. 2017) |
| Rutin | *Oryza sativa* | *Xanthomonas* *oryzae* pv. oryzae | ↑ resistance, primed expression of PGR | (Yang et al. 2016) |
| Rutin | *Tobacco* | *Ralstonia* *solanacearum* | ↑ resistance, primed expression of PRG | (Yang et al. 2016) |
| Rutin | *Arabidopsis thaliana* | *Pseudomonas* syringae pv. tomato | ↑ resistance, primed expression of PRG | (Yang et al. 2016) |
| Cinnamic acid | *Cucumber* | *Fusarium* *oxysporum* f. sp. *cucumerinum* | ↑ incidence of Fusarium wilt, oxidative stress  ↑ antioxidant enzyme activities | (Ye et al. 2006) |
| Naringenin | *Tobacco* | *Phytophthora nicotianae* | Antimicrobial activity against P. *nicotianae*, induced PR  ↑ expression of defense genes | (Sun et al. 2022) |
| Bxs, HCAA dimers, flavonoid, diterpenoid phytoalexins | *Grass family (Poaceae)* | Pathogen attack, herbivory | Accumulation of antimicrobial SM | (Ishihara 2021) |
| Pterostillbene, resveratrol and piceide | *Vitis vinifera* | *Botrytis cinerea* | ↑ in pterostilbene, resveratrol and piceide | (Morata and Loira 2019) |
| Aucuparin,  2'-Ha2'-Ma | *Aronia arbutifolia* | Fungal inoculation or natural infection | Biphenyl induction | (Kokubun and Harborne 1995) |
| Aucuparin, 2'-Ha2'-Ma4', Isoaucuparin | *Sorbus aucuparia* | Fungal inoculation or natural infection | Biphenyl induction | (Kokubun and Harborne 1995) |
| Glycyrrhizin | *Abrus precatorius* | *Aspergillus niger* and *Rhizopus stolonifer* | ↑ in glycyrrhizin productivity | (Dixit et al. 2011) |
| Artemisinin | *Artemisia annua* | *Penicillium oxalicum* | Induce oxidative stress | (Zheng et al. 2016) |
| Ajmalicine and Catharanthine | *Catharanthus roseus* | *Chrysosporium palmorum, Eurotium rubrum, Micromu- corisabellina* | ↑ synthesis of ajmalicine and catharanthine | (DiCosmo et al. 1987) |
| Asiaticoside | *Centella asiatica* | *Colletotrichum lindemuthianum* | Highest biomass accumulation | (Prasad et al. 2013) |
| Paclitaxel | *Corylus avellana* | *Chaetomium globosum* and *Paraconiothyrium brasiliense* | ↑ paclitaxel production | (Salehi et al. 2019) |
| Gymnemic acids | *Gymnema sylvestre* | *Aspergillus niger* | ↑ of gymnemic acid | (Devi 2011) |
| Phenylpropanoid and Naphtodianthrone | *Hypericum perforatum* | *Fusarium* *oxysporum*, *Phoma* *exigua* and *Botrytis* *cinerea* | ↑ in phenylpropanoid production | (Gadzovska Simic et al. 2015) |
| Menthol | *Mentha piperita* | *Aspergillus* *niger* | Stimulation of menthol production | (Chakraborty and Chattopadhyay 2008) |
| Rosmarinic acid | *Ocimum basilicum* | *Rhizophagus irregularis* | Elicitor of rosmarinic acid and antioxidant production | (Srivastava et al. 2016) |
| Ginsenoside | *Panax ginseng* | *Alternaria pa*nax | Ginsenoside synthesis of adventitious roots | (Hao et al. 2020) |
| Ginsenoside | *Panax quinquefolius* | *Trichoderma atroviride* | Rg3/Rh2 panaxadiol induction | (Biswas et al. 2016) |
| Ginsenoside and Anthocyanin | *Panax sikkimensis* | *Trichoderma harzianum* | ↑ expression of ginsenoside biosynthetic | (Biswas et al. 2018) |
| Abietic acid | *Leguminous / Pine trees* | Antipathogen | NA | (Elvin 1977) |
| Gikolides | *Ginko biloba* | AnttiBiotic | NA | (Jung et al. 2003) |
| Menthol | *Mentha spicata, Arabidopsis thaliana* | Antifungal | NA | (Lin et al. 2017) |
| Gossypol | *Gossypium* | Antipathogen / Antipest | NA | (Tian et al. 2019) |
| Sterols | *Asclepias syriaca* | Antiherbivore / Anti - insect | NA | (Abe 2007) |
| Leteolin, Chrysoeriol, Apigenin | *Apium graveolens / Thymus vulgaris* | AntiBiotic | NA | (Hou et al. 2004) |
| Quercetin,  Qaempferol,  Myrecetin | *Allium cepa / Allium porrum / Brassica oleracea* | Antipathogen | NA | (Jain et al. 2015) |
| Bergaptene | *Ammi majus* | Antibacterial | NA | (Aldulaimi 2017) |
| Gallic. Salicylic acids | *Berries / Nuts* | Antiviral | NA | (Ozçelik et al. 2011) |
| Plumbagin | *Plumbago spp./Drosophyllum* | Antimicrobial / Anti-insect | NA | (Komaraiah et al. 2003) |
| Emodin | *Higher plants* | Antiherbivore / Antipathogen / | NA | (Izhaki, 2002) |

**PRG*:*** pathogenesis-related genes; **PR**: pathogen resistance; **HCAA** :hydroxycinnamic acid amide; **Bxs**: Benzoxazinoids **2'Ha2'Ma** : 2' Hydroxyaucuparin 2'-Methoxyaucuparin ; **2'-Ha2'-Ma4'-Ma** :2'-Hydroxyaucuparin 2'-Methoxyaucuparin 4'-Methoxyaucuparin
